# *Mycobacterium tuberculosis* cell-wall and antimicrobial peptides: a mission impossible?

**DOI:** 10.3389/fimmu.2023.1194923

**Published:** 2023-05-17

**Authors:** Yolanda M. Jacobo-Delgado, Adrian Rodríguez-Carlos, Carmen J. Serrano, Bruno Rivas-Santiago

**Affiliations:** Biomedical Research Unit Zacatecas, Mexican Institute for Social Security-IMSS, Zacatecas, Mexico

**Keywords:** antimicrobial peptides, *Mycobacterium tuberculosis*, cell wall, tuberculosis, immune response, antimicrobial resistance

## Abstract

*Mycobacterium tuberculosis* (Mtb) is one of the most important infectious agents worldwide and causes more than 1.5 million deaths annually. To make matters worse, the drug resistance among Mtb strains has risen substantially in the last few decades. Nowadays, it is not uncommon to find patients infected with Mtb strains that are virtually resistant to all antibiotics, which has led to the urgent search for new molecules and therapies. Over previous decades, several studies have demonstrated the efficiency of antimicrobial peptides to eliminate even multidrug-resistant bacteria, making them outstanding candidates to counterattack this growing health problem. Nevertheless, the complexity of the Mtb cell wall makes us wonder whether antimicrobial peptides can effectively kill this persistent Mycobacterium. In the present review, we explore the complexity of the Mtb cell wall and analyze the effectiveness of antimicrobial peptides to eliminate the bacilli.

## Introduction

The *Mycobacterium tuberculosis* (Mtb) cell wall is probably the most complex membrane of all bacteria. The architecture of this complex wall extends to a massive core comprised of peptidoglycans covalently attached *via* a linker unit to a linear galactofuran and to several strands of a highly branched arabinofuran, and mycolic acids. Mycolic acids are oriented perpendicular to the membrane plane and provide a lipid barrier, which is the main feature of this bacteria (1). Several immune-evasion mechanisms conferred to Mtb, such as higher hydrophobicity, depend on its cell wall by masking pathogen-associated molecular patterns with phthiocerol dimycocerosates and inhibiting toll-like receptor 2 with sulfoglycolipids (2). These strategies inhibit the detection of bacteria and the activation of cytokine responses. They also recruit naive rather than microbicidal macrophages to sites of infection, preventing detection by targeting antigen presentation pathways of the adaptive immune system (2). *M. tuberculosis* uses several components of the cell wall to alter the processing and availability of peptide antigens for MHC class I and MHC class II. Furthermore, these cell wall-derived molecules can inhibit innate immunity processes such as autophagy (3, 4).

During primary infection, the first cells to encounter Mtb are lung epithelial cells (EpCs) and alveolar macrophages which are capable of sensing and mounting an immune response *versus* Mtb, mainly through a wide variety of antimicrobial peptides. Most of these peptides display conserved properties including amphipathicity, cationicity and a small molecular size (12-50 amino acid residues) (5). Whether or not these short cationic peptides can disrupt this thick and impermeable wall will be discussed herein.

Although tuberculosis [TB] is a preventable and treatable disease, it remains an important threat to public health. Transcriptomic analysis has shown that MDR-Mtb is capable to modify acetylation/methylation patterns in macrophages and lymphocytes infected, leading to oxidative stress and premature cellular aging, which correlates with higher intracellular survival and dissemination. Besides, some mycobacterial products play epigenetic changes promoting DNA methylation or altering the expression of non-coding RNAs to upregulate the immune response activation (6, 7).

Despite the growing effort of the global health community to eradicate tuberculosis, in 2021 a total of 1.5 million people died from TB. In fact, Mtb was the second leading infectious killer after COVID-19. Due to vaccination and management of the pandemic, TB is profiling to return as the leading cause of mortality. This time, however, it will be even stronger as all healthcare resources were directed to the containment of SARS-Cov2 during the COVID-19 pandemic and important TB control programs, including vaccination, multidrug-resistant surveillance, and direct observed therapy, were ignored (DOT) (8, 9). Nowadays, drug-resistant tuberculosis is a major public health challenge, and it poses a significant threat to global efforts to control TB. The treatment of drug-resistant TB is complex, lengthy, and expensive, and it often requires the use of toxic and poorly tolerated drugs. The emergence of extensively drug-resistant TB (XDR-TB) further complicates the situation, as there are limited treatment options available. There is an urgent need for new and more effective therapies to combat drug-resistant TB, and antimicrobial peptides are a feasible option to shorten and improve the conventional therapy to address this pressing public health challenge.

*M. tuberculosis* spreads from person to person almost exclusively by aerosolized particles. Nearly 90% of Mtb-infected individuals spontaneously control infection and eliminate mycobacteria (10, 11), while the remaining percentage of infected individuals contain the bacteria in a granuloma. Interestingly, numerous individuals living in densely populated areas prone to TB seem to be resistant to Mtb infection. They may presumably have immediate elimination of Mtb by innate phagocytes, epithelial cells, soluble antimicrobial molecules, innate invariant T cells, and natural killer cells, located in the mucosa and alveoli of the bronchial pulmonary airway mucosa and alveoli. They appear to have never been Mtb-infected as they are found to be negative for tuberculin skin test reaction and show an absence of granulomas. The resistance of such individuals may suggest the capability of innate immunity as a major natural effector against Mtb (12).

The defining characteristic of Mtb that is atypical of other bacteria is the complexity of its cell wall, which is associated with pathogenesis and provides a barrier against antibiotics and the immune response of the host (13, 14).

## Mycobacterium tuberculosis: a complex cell-wall

**Figure 1:**
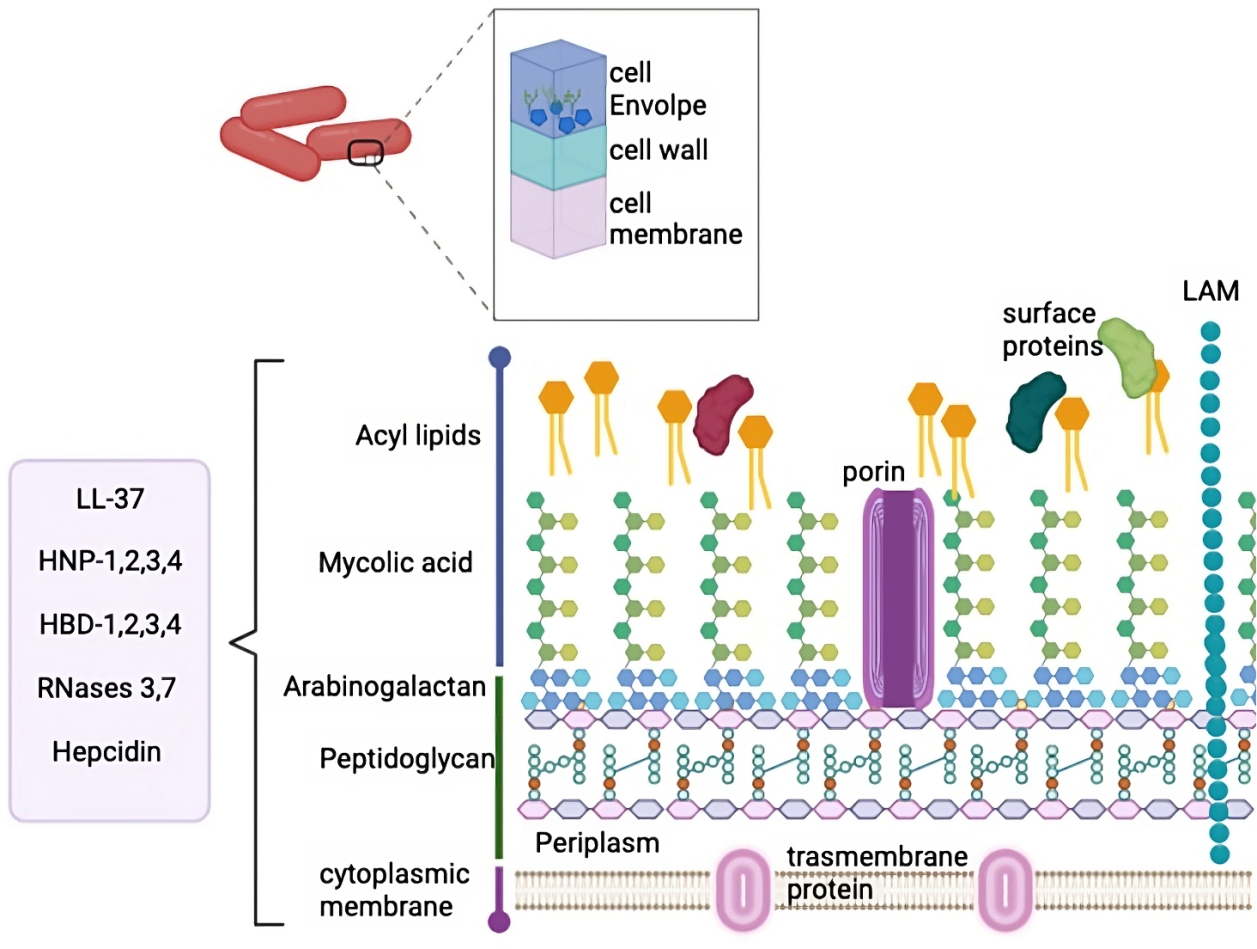
*M. tuberculosis* cell wall. MTb cell wall is composed by several complex layers, including cell envelope, cell wall and cell membrane. These layers are composed by multiple lipids and peptidoglycan. The peptides that have been described to interact with this complex cell wall are mentioned in the box.

The *mycobacterium* cell wall is a unique characteristic of this bacilli described for the first time in 1995 (15, 16). This structure is composed of three segments: the plasma membrane, the cell wall core, and the outermost layer (illustrated in **Figure 1**) (17). Around 60% of the mycobacterium mass is constituted by complex lipids, giving it extreme hydrophobicity (18, 19). The plasma membrane is similar to other bacteria and provides structural integrity and support (18). The plasma membrane is surrounded by a highly cross-linked peptidoglycan (PG) layer and forms covalent complexes with arabinogalactan (AG) (18). The PG organization is dynamic and confers thickness and a physical barrier that protects bacteria from potential damage in the microenvironment (20). Beyond PG, AG is also an essential component. In Mtb, the length of the AG polysaccharide affects the shape and hydrophobicity of the bacilli membrane (21). Then, superficial AG ends are esterified with unusually long high molecular weight fatty acids called mycolic acid and represent the major fraction of the cell wall. Mycolic acid is strongly hydrophobic and forms a shell around the bacilli to confer protection against hydrophilic antibiotics, oxidative damage, and complement deposition (22).

The cell wall is also composed of mycolic acids interleaved with free lipids which are essential for viability. Teichoic acid, another cell wall component, binds to the plasmatic PG, thus conferring high stability and giving rise to a compact structure (17, 18). The outer membrane is mainly composed of mycolic acid and complex glycolipids, such as trehalose monomycolate, trehalose dimycolate (TDM), phospholipids, glycopeptidolipids, phthiocerol dimycocerosate, phosphatidylinositol mannosides, phthioceroldimycocerosates, lipomannan, lipoarabinomannan (LAM) and sulfolipids (18).

Mycobacterial glycolipids interact with receptors in host cells to promote bacilli internalization and modulate the immune response, allowing bacterial replication (18). The glycolipid TDM is a virulence factor known as the cordon factor, and it accumulates in a cordon-like fashion on the bacilli´s surface (22). The cell wall of *Mycobacterium* is a complex structure associated with intrinsic resistance to clearance in infected cells with the lipid barrier functioning as a shield in harsh environments (22). The composition of the cell wall and hydrophobicity confer low permeability (20). Furthermore, Mtb has efflux pumps (15) and can regulate its cell wall components by proteolysis, and the proteases involved are considered virulent determinants (23).

Overall, the high resistance and virulence of the mycobacterial cell wall are provided by the complex relations and intertwining between their components. However, it is well-documented that some antimicrobial peptides (AMPs) can damage and disrupt the mycobacterial membrane.

## *Mycobacterium tuberculosis* meets antimicrobial peptides

**Figure 2:**
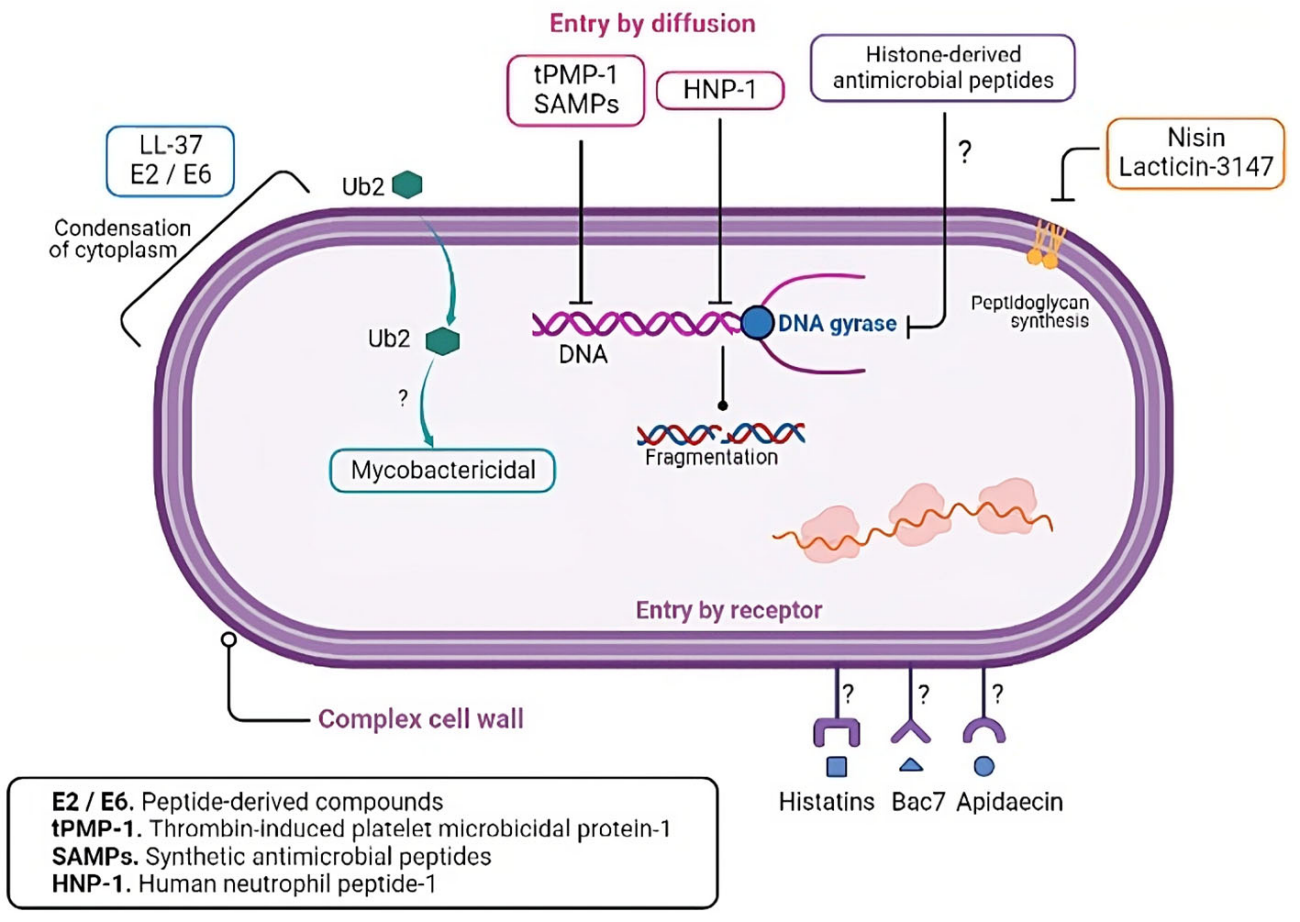
Mechanisms of action of the antimicrobial peptides. Antimicrobial peptides can exert their lytic effects directly on the membrane or over intracellular targets. Regarding the membrane action, after the initial electrostatic interaction, AMPs accumulate and form different pores structures. The pores can be transmembranal and are classified into the barrel and toroidal models. In the barrel model, AMPs insert perpendicularly in the lipid bilayer, while in the toroidal model, AMPs induce a local curvature to insert and form the pore. Moreover, in the carpet model, AMPs can cover the surface of the membrane and form a carpet that destabilizes it, producing ruptures and micelle formation. In the aggregate model, AMPs aggregate on the membrane surface and induce similar results as those in the carpet model. In addition, some peptides translocate the membrane to bind intracellular targets such as enzymes or components needed in vital processes, inhibiting ARN, DNA, or protein synthesis.

The search for new antimicrobial molecules it is not recent, and some of these studies have highlighted the use of peptides. During the mid-20th century, the preliminary research on AMPs described their ability to kill bacteria. During World War II, the antimicrobial agent gramicidin D successfully treated infected wounds (24). Since then, nearly 3400 AMPs have been described (https://aps.unmc.edu/). These peptides include synthetic peptides and those of bacteria, bacteriophages, plants, humans, and other mammals (24). AMPs have been classified according to their physicochemical properties: net charge, structure, and solubility. Most AMPs contain both hydrophobic and hydrophilic side chains that enable the solubility of these molecules in most organic media. The positive charge allows them to interact with most microbial membranes and the amphipathic properties make the AMPs insert into the membrane, leading to pore formation which causes cell death by osmotic shock (**Figure 2**). AMPs can be also divided based on their primary structures such as linear peptides, and some of the most important classes of AMPs in these groups are cecropin, magainin, the human cathelicidin LL-37 and their derivatives, and proline rich AMPs. Secondary structures may have four architectures that include α-helical, β-stranded due to the presence of disulfide bonds, β-hairpin or loop due to the presence of cyclization of the peptide chain, and extended non-classical peptides (25). Based on their antimicrobial mechanism, these peptides are classified into membrane acting and non-membrane acting peptides (26). Whereas membrane acting peptides provoke membrane disruptions, non-membrane peptides are capable of translocating across the membrane without causing damage. Few antibacterial peptides create trans-membrane pores on the target membrane and include defensin, melittin, magainins, and LL-37. AMPs such as buforin II, dermaseptin, HNP-1, pleurocidin, indolicidin, pyrrhocoricin, and mersacidin translocate across the cell membrane and disrupt normal cell functioning (27). AMPs also block processes like protein synthesis, nucleic acid synthesis, enzymatic activities, and cell wall synthesis (28). The broad properties of peptides make them one of the best possible alternatives for controlling TB infections.

The first approach to investigating the activity of AMPs on Mtb bacilli was reported in cytotoxic T lymphocytes. In 1999, pioneer research observed that granulysin killed Mtb at 72 hours post-exposure in a dose-dependent manner. Furthermore, they described that *in vitro* the association perforins-granulysin is needed to provide access to intracellular mycobacteria (29). A more direct approach to demonstrate the AMPs protective effect on Mtb was assessed using the human neutrophil peptide (HNP)-1 (30, 31). These reports described that HNP-1 inhibited Mtb growth. Similarly, porcine leukocyte protegrin showed a 99% reduction in colony-forming units (CFUs) of Mtb, even in clinical isolates (30). Other studies have shown that the application of HNP-1 inhibited intracellular mycobacteria growth in infected macrophages after 3 days of treatment (31). Indeed, HNP-1 can be taken up from apoptotic neutrophils since the phagocytosis of these apoptotic neutrophils by macrophages leads to a decrease in the viability of intracellular bacilli. During this process, the granule contents travel to early endosomes and co-localize with mycobacteria (32).

Some years later, *in vitro* evaluation of protegrin (PG)-1 and beta-defensin (HBD) -1 activity showed not only the effectivity of both peptides in TB infections, but also that the combination of PG-1 or HBD-1 with isoniazid significantly reduced Mtb growth when compared to peptides or isoniazid alone (33). In addition, our group compared the efficacy between LL-37, cathelicidin-derived antimicrobial peptide (CRAMP) and the synthetic peptides E2, E6, and CP26, which had moderate antimicrobial activities against Mtb. However, intratracheal therapeutic application of these peptides in a mouse model with TB reduced pulmonary mycobacterial burden after 28 days of treatment (34). Other interesting approaches using cellular synthesis of HBD-2 *via* highly efficient human macrophage mRNA transfection improved mycobactericidal and mycobacteriostatic activity by macrophages, inhibiting intracellular growth by 50% (35). This suggests the importance of this defensin for eliminating mycobacteria in macrophages.

The airway epithelial cells are the first cells to interact directly with Mtb and pioneer studies demonstrated that stimulation of these cells with mannosylated lipoarabinomannan (manLAM) promoted HBD-2 gene expression (36), while LL-37 can be induced by lipopolysaccharide (LPS), Mtb DNA, and lipoarabinomannan (LAM) through toll-like receptors (37). AMPs produced during EpC infection and macrophage infection are released into the extracellular space; however, ultrastructural studies have revealed that these peptides can associate with intracellular mycobacteria, thus promoting membrane disruption (36). This finding suggests that defensin induction contributes to an efficient bacterial control through cell wall interaction.

Although mice are not a natural reservoir for Mtb, this model has served to unveil several immune response issues, including those related to AMPs. The mouse strains with lower levels of defensins are more susceptible to developing active pulmonary TB, while the B6D2F1 mouse strain, which has higher defensin expression values, develops latent TB infection (38). Similarly, the use of mice models and *cramp*−/− knockout Balb/c mice has helped to understand the role of cathelicidin in TB (39, 40). The Cramp−/− mice develop more pneumonic areas, lower inflammatory cytokines levels (IFN-γ, IL12p40, and TNFα), and higher Mtb intracellular growth than wild-type mice (41).

Accumulated evidence over several decades has collectively shown the importance of AMPs in eliminating Mtb both *in vivo* and *in vitro* and points to their potential use as ‘the new era antibiotics’.

## Do AMPs really interact with Mtb cell wall?

The interaction of AMPs with the mycobacterial cell wall is different from that of other bacteria membranes because of their composition and structure. Several authors described a lower antimicrobial activity of AMPs over mycobacteria in comparison with Gram-negative or positive bacteria. The direct interaction of AMPs with the mycobacterial cell wall is imperative to increase permeability through the formation of pores leading to cell death (28). Several reports have observed more than one mode of action (28), and experimental evidence describes the presence of specific AMP binding sites on the cell wall. For instance, AMPs bind to PG in a time-dependent fashion and require a threshold concentration of AMPs (42).

**Figure 3:**
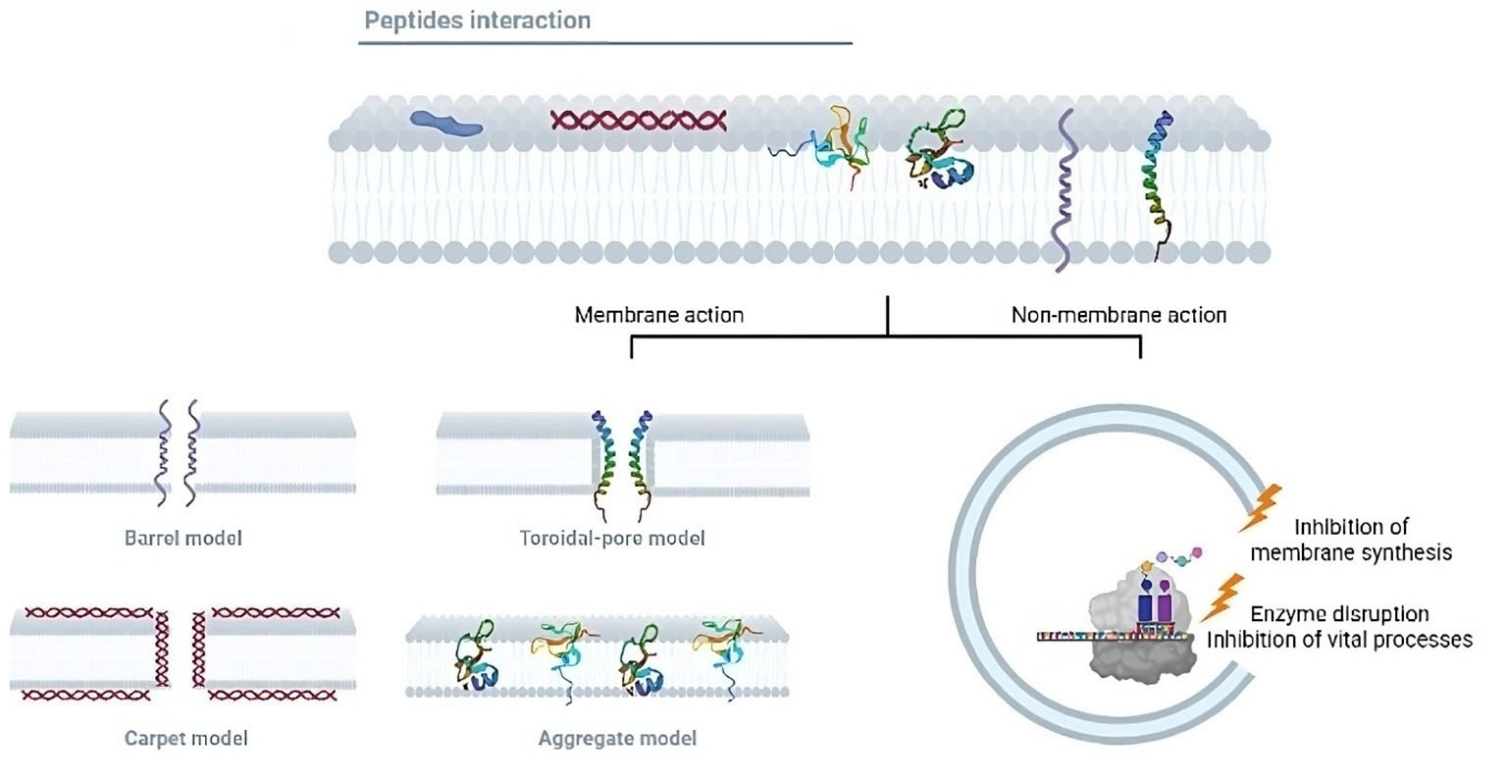
AMPs intracellular targets in *M. tuberculosis*. Besides the cell wall, several studies have shown that AMPs have intracellular targets in *M. tuberculosis*, including nucleic acids, gyrases, and ribosomes.

Even though the AMPs expressed in the lungs have been tested against different strains of mycobacteria, only some demonstrated direct interaction with the cell wall (**Figure 3**). For example, *in vitro* studies showed that LL-37 induced a dose-dependent reduction in CFU/mL of Mtb H37Rv (43, 44). Immunofluorescence microscopy analysis shows the uptake of LL-37 by macrophages. The authors observed the colocalization of LL-37 with *M. smegmatis* and BCG-containing vesicles. Concomitantly, they reported the colocalization of lysosomes and pathogenic mycobacteria-containing phagosomes (44). Ultrastructural analysis also revealed that LL-37 and CRAMP induced a homogeneous increase in the electron-lucent cell wall surrounded by a thin electron-dense rim (34). These observations indicated that the cell wall is an important target of LL-37.

Alpha-defensins are a class of peptides with important antimycobacterial activity. For instance, HNP-1 is active against Mtb with MIC values of 0.8 μg/mL and 2.5 μg/mL (31). HNP-1 is released into the extracellular environment by neutrophils and is taken up by macrophages to kill intracellular pathogens (31). Moreover, scanning electron micrographs revealed that HNP-1 induces wart formation on the surface of Mtb (30). Another study found that HNPs act according to their composition since the adsorption of HNP-1 on the negatively charged phospholipids monolayer reaches the equilibrium in less than 2 min, whereas it takes more time in the case of HNP-2 and 3 (45). These findings suggest that HNP-1 penetrates a lipid monolayer and interacts with phospholipids.

HBD-1 showed a reduction in CFU at 128 μg/ml (33), while HBD-2 had antimicrobial activity at 1.5 μM (35). Furthermore, macrophages infected with Mtb were immunostained for HBD-2 and these peptides were found associated with intracellular Mtb rather than in the cytoplasm (35). We similarly observed the adhesion of HBD-2 to the Mtb cell wall in infected epithelial cells by electron microscopy (36). On the other hand, up-regulation of HDB-3 and -4 was reported to be effective in Mtb MDR-infected mice (46). This experimental evidence suggests that mycobacteria infections may also be amenable to β-defensins by direct cell wall interactions.

Human antimicrobial RNases are small secretory proteins that belong to the RNase superfamily. RNase7, together with RNase3, can eradicate mycobacteria *in vitro* (47) and recent electron microscopy results showed that RNase 7 could attach to the cell wall of intracellular mycobacteria (48). In contrast, hepcidin is an antimicrobial peptide produced in mouse bone marrow-derived macrophages by mycobacteria infection (49), though hepcidin has low activity against Mtb. Confocal microscopy analysis, however, showed that hepcidin was localized to mycobacteria-containing phagosomes and had direct antimicrobial activity (49).

We summarized the AMPs’ interaction with mycobacterial membranes and CFUs load in the respective experiments in **Table 1**. These reports have demonstrated an accumulation of AMPs inside the bacterial cells after permeabilization. This internalization contributes to the killing of bacteria by intracellular targets.

**Table 1:** Antimycobacterial peptides and peptidomimetics from diverse origin and their reported interaction with the cell membrane of pathogenic *Mycobacterium tuberculosis* (H37Rv and/or clinical isolates).

PEPTIDE	AMP ORIGIN	MECHANISM ON MYCOBACTERIAL MEMBRANE	EFFECT ON Mtb VIABILITY	REFERENCES
HNP-1	Azurophilic granules of human polymorphonuclear neutrophils	Disrupts microbial cell membrane and inhibits cell wall biosynthesis	Inhibit Mtb-growth in antimicrobial assays (MIC determination). Colony forming units (CFU) assays. Reduction of mycobacterial load in macrophages infected *in vitro*	(30, 50, 51)
HNP-2	Azurophilic granules of human polymorphonuclear neutrophils	Disrupts the microbial cell membrane	Inhibit Mtb growth in MIC determination, CFU assays, and reduction of the mycobacterial load in macrophages infected *in vitro*	(45, 51)
HNP-3	Azurophilic granules of human polymorphonuclear neutrophils	Disrupts the microbial cell membrane	Inhibit Mtb growth in MIC determination, CFU assays, and reduction of the mycobacterial load in macrophages infected *in vitro*	(45, 51)
HBD-1	Human leukocytes and epithelial cells	Disrupts the microbial cell membrane	Inhibit Mtb-growth in MIC determination	(33, 45)
HBD-3	Human leukocytes and epithelial cells	Inhibits cell wall biosynthesis	Inhibit Mtb-growth in MIC determination	(52, 53)
HBD-4	Human leukocytes and epithelial cells	Disrupts the microbial cell membrane	Inhibit Mtb-growth in MIC determination	(45)
LL-37	Human macrophage and neutrophils	Pore formation	Reduction of mycobacterial load in macrophages infected *in vitro*	(40, 54)
Human beta-defensin-1 (HBD-1)	Human leukocytes and epithelial cells	Formation of cation-selective channels on bacterial membrane	Inhibit Mtb-growth in MIC determination	(33)
Ub2	Human lysosome	Disrupts microbial cell membrane	Killing mycobacteria in the lysosomal compartment	(55, 56)
Dermcidin	Sweat glands of human	Inhibits cell wall biosynthesis and binds to bacterial envelope	Not determined	(57, 58)
NP-1	Rabbit (*Oryctolagus cuniculus*) leucocytes	Disrupts the microbial cell membrane	Inhibit Mtb-growth in CFU assays	(30)
Protegrin-1 (PG-1)	Porcine (*Sus scrofa*) leukocytes	Formation of cation-selective channels on bacterial membrane	Inhibit Mtb-growth in MIC determination	(33)
mCRAMP	Macrophage of mouse (*Mus musculus*)	Interferes with inner membrane	Inhibit Mtb-growth in MIC determination. Reductions in lung bacilli in mouse TB experimental model	(34, 59, 60)
Indolicidin	Bovine neutrophils	Disruption of the bacterial membrane	Inhibit Mtb-growth in MIC determination	(61)
VpAmp2.0	The venom glands of the Mexican scorpion Vaejovis punctatus	Target membrane proteins	Inhibit Mtb-growth in MIC determination	(62)
Pin2	The venom of the African scorpion Pandinus imperator	Pore formation	Inhibit Mtb-growth in MIC determination	(63)
BICTcu5	The skin secretion of the Indian frog *Clinotarsus curtipes*	Alteration of the mycobacterial membrane (thinning, pore formation, and altered curvature)	Reduction of mycobacterial load in macrophages infected *in vitro*	(64)
Teixobactin	β-*proteobacterium eleftheriaterrae*	Inhibit cell wall synthesis components (peptidoglycan and teichoic acid)	CFU assays	(65)
Nisin A	*Lactococcus lactis*	Inhibit peptidoglycan synthesis and pores formation	Inhibit Mtb-growth in MIC determination	(66)
Lacticin 3147	*Lactococcus lactis*	Inhibit cell wall synthesis (peptidoglycan) and pores formation	Inhibit Mtb-growth in MIC determination	(66)
GranF2	Synthetic, derived from granulysin bactericidal protein	Disrupts the microbial cell membrane	Inhibit Mtb-growth in MIC determination. Reduction of mycobacterial load in macrophages infected *in vitro*	(67–69)
G-13	Synthetic, derived from granulysin bactericidal protein	Disrupts the microbial cell membrane	Inhibit Mtb-growth in MIC determination	(67)
D-LAK120	Synthetic	Pore formation	Inhibit Mtb-growth in MIC determination	(70)
M(LLKK)2M	Synthetic	Membrane lytic-mechanism	Inhibit Mtb-growth in MIC determination	(71)
WKWLKKWIK	Synthetic	Pore formation and disrupting the organization of the lipid bilayer	Inhibit Mtb-growth in MIC determination	(72)
Cationic peptoid (1-C134mer)	Synthetic	Micellization and disrupting the organization of the lipid bilayer	Inhibit Mtb-growth in MIC determination	(73)
LLKKK18	Synthetic	Pore formation	Reduction of mycobacterial load in macrophages infected *in vitro*	(74)
1-C13	Synthetic	Formation of bacterial pores	Inhibit Mtb-growth in MIC determination and Alamar blue assay (MABA)	(73)
CAMP/PL-D	Synthetic	Formation of bacterial pores	Inhibit Mtb-growth in MIC determination	(72)
CP26	Synthetic	Disruption of bacterial cell wall	Inhibit Mtb growth in MIC determination) and Reductions in lung bacilli in mouse TB experimental model	(34, 75)
D-LL37 (D1 and D5)	Synthetic	Formation of bacterial pores	Inhibit Mtb growth in antimicrobial assays (MIC determination)	(43)
E2 and E6	Synthetic	Disruption of bacterial cell wall	Inhibition of Mtb growth in the determination of MIC and reductions in lung bacilli in the mouse TB experimental model	(34, 75)
Pandinin 2 variants	Synthetic	Disruption of the bacterial cell membrane	Inhibition of Mtb growth in MIC determination and growth inhibition curves	(63)
RN3, RN6, RN7 (1-45)	Synthetic	Disruption of bacterial cell wall	Intracellular macrophage killing	(47)
X(LLKK)2X: II-D, II-Orn, IIDab and IIDap	Synthetic	Formation of bacterial pores	Inhibit Mtb-growth in MIC determination and reduced the intracellular bacterial burden in mouse macrophage cell line RAW 264.7	(47)
IDR-HH2	Synthetic	Disrupts thinning and budding of the cell wall.	Inhibit Mtb growth in MIC determination and Reductions in lung bacilli in mouse TB experimental model	(75)
Gran1	Synthetic	lethal distortions of the cell wall	Inhibition of Mtb growth in macrophages	(76)

Adapted and updated from (77–79).

## Mycobacterial intracellular targets of AMPs

AMPs have several mechanisms of action besides their lytic capacity (**Table 2**). It is well-documented that AMPs can interact with intracellular components to prevent microorganism replication or survival (114–116) (illustrated in **Figure 3**). AMPs cause pore formation in the membrane leading to cell death and the hydrophobicity, along with their small size, allows them to diffuse through the membrane, though the exact mechanism is unknown. Some peptides such as histatins, Bac7, and apidaecin, have stereospecific characteristics that allow them to translocate inside cells through specific receptors (114). The conjugation of antimicrobial peptides with conventional antibiotics enhances susceptibility to the second, probably because the peptide facilitates the translocation of the conjugated molecule (117). Moreover, there are peptides with more than one mechanism that are concentration or time dependent. Thrombin-induced platelet microbicidal protein-1 (tPMP-1), for example, shows lytic activity in higher concentrations within minutes after exposure, and lower concentrations have delayed effects in 1 to 2 hours post-exposure such as inhibition of protein or DNA synthesis (118).

**Table 2:** AMPs with antitubercular activity with targets different or additional to cell membrane disruption/synthesis inhibition.

PEPTIDE	AMP ORIGIN	MECHANISM	REFERENCES
LL-37	Human macrophage and neutrophils	Immunomodulatory activity	(40, 73)
Proregion of human hepcidin	Macrophages and liver hepatocytes of human	Inhibits the growth of bacteria *via* preventing the release of recycled iron	(40)
HCL2	Part of human cytochrome C oxidase subunit-3 proteins	Interacts with the culture filtrate protein	(80)
RN3, RN6, RN7 (1-45)	Secreted by eosinophil secondary granules of human	Cell agglutination, intracellular macrophage killing	(47)
HNP-1	Azurophilic granules of human polymorphonuclear neutrophils	Inhibits DNA synthesis	(30, 50, 51, 81)
HBD-2	Human leukocytes and epithelial cells	Inhibits bacterial growth *via* vitamin D pathway.	(35, 53, 82, 83)
Hepcidin-25	Macrophages and liver hepatocytes of human	Inhibits the growth of bacteria *via* preventing the release of recycled iron	(49, 84)
Dermcidin	Sweat glands of human	RNA and protein synthesis inhibition	(57, 58, 85)
PR-39	Porcine (*Sus scrofa*) leucocytes	Inhibition of DNA and protein synthesis	(86)
Trichoderin A	Marine sponge derived fungus of *Trichoderma* sp.	Inhibits ATP synthase	(87, 88)
Trichoderin B	Marine sponge derived fungus of *Trichoderma* sp	Inhibits ATP synthase	(87)
Callyaerin A	Marine sponge *Callyspongia aerizusa*	Not determined	(89, 90)
NZX	Derived from fungus plectasin protein	Target intracellular bacteria without lysing cells and Immunomodulatory effect	(91)
Lassomycin	*Lentzea kentuckyensis*	Binds to ClpC1P1P2 and cause uncoupling ATPase from proteolytic activity	(92)
Capreomycin	Streptomyces *capreolus*	Inhibits protein synthesis	(93–95)
Viomycin	*Streptomyces puniceus*	Inhibits protein synthesis	(94, 96)
Ecumicin	*Nonomuraea* sp. MJM5123	Inhibits the action of ClpC1	(97, 98)
Wollamide A	*Streptomyces nov*. *sp* (MST-115088)	Not determined	(99)
Wollamide B	*Streptomyces nov*. *sp* (MST-115088)	Not determined	(99)
Rufomycin I/llamycin A	*Streptomyces* sp. (MJM3502) *Streptomyces atratus* (NRRL B-16927)	Inhibits the action of ClpC1	(99–101)
Cyclomarin A	*Streptomyces* sp. (CNB-982)	Inhibits the action of ClpC1	(102–105)
E50-52	*Enterococcus faecalis*	Formation of E50-liposome-complex without damaging the cell membrane	(106, 107)
Calpinactam	*Mortiella alpine* FKI-4905	Not studied	(108–110)
LLKKK18	Synthethic, derivative of LL-37	Immunomodulatory activity	(74, 111)
Synthethic AMPs (SAMPs- Dma)	Synthetic	DNA binding	(112)
G-13	Synthetic, derived from granulysin bactericidal protein	Induces apoptosis of mammalian cell	(67)
GranF2	Synthetic, derived from granulysin bactericidal protein	Induces apoptosis of mammalian cell	(67–69)
IP-1	Synthetic	Autophagy activation and TNF-α secretion	(113)

Adapted and updated from (77–79).

Although there is no evidence of the mechanism involved, it has been suggested that AMPs can translocate the complex cell wall of mycobacteria. For instance, synthetic ubiquitin-derived peptide Ub2 shows mycobactericidal activity, but when the peptide was tested against *Mycobacterium* with reduced membrane permeability, it lost its ability to cause cell death (119). This evidence suggests that Ub2 activity depends on its ability to enter cells and has no direct effect on the membrane.

It is well documented that Mtb is susceptible to HNP-1, and the membrane disruption is dose dependent (30, 120). A morphological evaluation using scanning electron microscopy revealed that a low dose of HNP-1 induces less damage to the membrane, and this correlates with a decrease in the CFUs as well (120). HNP-1 was reported to bind to mycobacterial genomic DNA with the consequent disrupted DNA biosynthesis (81). In summary, DNA is proposed as an intracellular target of HNP-1 against Mtb, and HNP *in vitro* induces DNA breaks (121). This mechanism deserves further research in the context of the interaction with Mtb.

Synthetic antimicrobial peptides (SAMPs) also showed a selective ability to translocate in Mtb and not to other bacteria membranes such as *E. coli*. SAMPs also cross the mammalian membranes in infected cells to reach the bacilli, and they do not have toxic effects on the host cells. Upon translocation, SAMPs interact directly with genomic DNA in Mtb, impairing DNA-dependent processes which lead to cell death (112).

Nisin and lacticin-3147 are AMPs produced by microbial fermentation and both inhibit peptidoglycan synthesis in non-pathogenic *M. smegmatis* and *M. bovis* as well as in clinically relevant mycobacteria. Both peptides must bind to the peptidoglycan precursor, and although not an intracellular target, it is located in the inner membrane (66).

**Figure 4:**
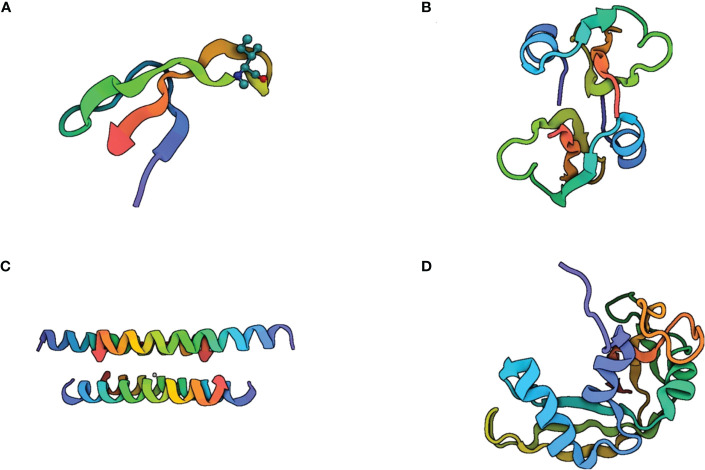
Structure of the most studied antimicrobial peptides in tuberculosis immunopathogenesis. **(A)** Human Neutrophil Peptide-1 (HNP-1) is a small cationic peptide composed of 37 amino acid residues. It is predominantly α-helical in structure, with a helix-hinge-helix motif. The helices are stabilized by hydrogen bonding between the peptide backbone and the side chains of certain amino acids, such as glutamic acid and lysine. HNP-1 also contains two disulfide bonds that help to maintain the peptide’s structure. **(B)** human β-defensin-2 is a 41-amino acid peptide with a β-sheet structure. It consists of three β-strands connected by two loops, forming a hairpin-like structure. The β-strands are stabilized by hydrogen bonds between the peptide backbone and the side chains of certain amino acids, such as asparagine and threonine. Human β-defensin-2 also contains three disulfide bonds that contribute to its stability. **(C)** Cathelicidin LL-37 is a 37-amino acid peptide that can adopt several different conformations, including α-helical, β-sheet, and random coil structures. The α-helical conformation is believed to be important for the peptide’s antimicrobial activity, while the β-sheet structure may play a role in its ability to interact with cell membranes. **(D)** RNAse 7 is a small cationic peptide composed of 128 amino acid residues. It has an α-helical structure with two disulfide bonds that help to stabilize the peptide’s conformation. The α-helices are amphipathic, this allows RNAse 7 to interact with and disrupt bacterial cell membranes.

Beyond the lytic activity of cathelicidin LL-37 and the peptide-derived compounds E2 and E6, an electron microscopy analysis showed that these peptides induce condensation of the cytoplasmic components in Mtb. This effect could be a consequence of the interaction between the peptide and the membrane (34). The most studied antimicrobial peptides within tuberculosis immunopathogenesis are HNP-1, HBD-2, LL-37, and RNAse7 (**Figure 4**).

There are other existing antibacterial mechanisms described for Gram-negative and Gram-positive bacteria, and although not yet identified in Mtb, they constitute a putative antimycobacterial mechanism. For example, histone-derived antimicrobial peptides have potential growth inhibition like that of LL-37. This property is based on the inhibition of DNA gyrases that interrupt bacterial division (122).

## Possible failures in the use of antimicrobial peptides as therapeutics

Although antimicrobial peptides are potentially good candidates for further use in the treatment of pathogen-associated diseases, there are several factors that should be considered prior to their use. One of the main factors is the complexity of the Mtb cell wall, which is a formidable barrier not only against drug therapies but also to AMPs. This wide resistance shown by Mtb is due to its complex cell wall, the presence of several enzymes, and its high mutation rates (77). The thickness and high presence of lipids compared to other bacteria and the tangible natural resistance of some Mtb strains to AMPs (123) make the task of AMPs even harder.

Another important consideration is the broad immunomodulatory activity these peptides provide. In a model of TB transmission by long cohabitation of infected and noninfected mice, the noninfected mice were treated with AMPs to determine the potential capacity of defensins to prevent the infection. Whereas peptides significantly decreased the bacterial burden, the inflammatory area evidently increased, suggesting that AMPs promoted also important pulmonary inflammation (124). Similarly, previous studies have reported that the angiogenic factor VEGF is secreted by Mtb-infected macrophages, promoting the formation of blood vessels. Inhibition of angiogenesis, *via* VEGF inactivation, abolishes the spread of mycobacteria from the infection site. It is interesting to note that an elevated level of VEGF was found in TB patients, suggesting that mycobacteria take advantage of the formation of new blood vessels to disseminate within the lung and to other organs (125). Several research groups have demonstrated the role of some AMPs in the promotion of angiogenesis. A previous study depicted that HBD-3 enhanced the secretion of angiogenic growth factors such as fibroblast growth factor, platelet-derived growth factor, and vascular endothelial growth factors (126). Similarly, LL-37 exerted *via* activation of endothelial cells and subsequent prostaglandin E2 (PGE2) biosynthesis important angiogenic activity (127). The use of AMPs as a therapeutic for pulmonary TB could thus induce a dissemination of mycobacteria that worsens the clinical outcome.

Another important gap for the use of AMPs in TB is that the pathological hallmark of TB is the granuloma, which is an organized and localized aggregate of immune cells consisting of macrophages, lymphocytes, and other host immune cells. The formation of granulomas is crucial to control and contain infection, but granulomas can also contribute to early proliferation and dissemination of Mtb (128). The granuloma is a crucial structure that shapes antibiotic access to the mycobacterium contained within. Antimycobacterial antibiotics are first transported to their site of action *via* the host vasculature; then, they must cross the cellular layers that compose the granuloma and finally access Mtb in the granuloma. The diffusion of the antibiotics will depend on the granuloma complexity, which varies enormously even within the same lung, frequently delivering a subclinical concentration of antibiotics leading to antibiotic resistance (129). Similar problems can be developed with the use of AMPs as therapeutic; it is likely that mycobacteria develop acquired resistance to AMPs and generate new mutants with higher levels of resistance after long-term use. In addition, there can also be potential risks in the long-term administration of AMPs due to their intrinsic properties such as immunogenicity, cytotoxicity, hemolytic activity, and other adverse effects (77).

Although there are many successful peptides tested against Mtb both *in vivo* and *in vitro*, and some of them in clinical trials (130–135), there is still a lack of sponsorship from pharmaceutical companies due to the high cost of synthesis and production of AMPs, and because of the aforementioned issues that need to be cleared up. Although the outlook seems dark, some approaches could allow us to circumvent these problems. For example, the use of other delivery methods such as nanoparticles which can directly enter infected cells, or the synthesis and design of smart peptides, which would be with an activity directed towards either a modulator profile or a merely antimicrobial profile. To avoid the emergence of multidrug-resistant strains, it will always be advisable to apply AMPs in combination with another drug. In the end, it would have to be evaluated whether the use of AMPs would reduce treatment time or be used in the company of antibiotics in conventional treatment regimens, or if peptides would be used accordingly for the desired effects: antimicrobial and immunomodulatory. As can be seen, there is still much research to be done and as quickly as possible, since the emergence of antibiotic-resistant strains is growing every day.

## *M. tuberculosis* strikes back and resistance to AMPs: what do we know so far?

The Mtb cell envelope has distinct lipopolysaccharides and glycolipids that play a critical role in Mtb survival and pathogenesis. The disruption of pathways involved in the assembly of the Mtb cell envelope has been suggested as the main target of antitubercular agents (78). Antimicrobials targeting bacterial membranes are generally known to be lipophilic and actively react to the lipid bilayer of the bacterial membrane (136). Researchers have proposed that the way AMPs interact with the cell membrane would define resistance and synergy mechanisms in four stages (137). The first stage is when the peptides are outside of the cell, exposed to peptidases (138). The selection of microorganisms with mutated extracellular peptidases, enhanced extracellular peptidase synthesis pathways, improved transport of peptidases to the extracellular space, or a combination of these elements could be a Mtb defense against AMPs. The second stage of the interaction is at the cell wall or outer membrane of the cell. This structure contains charged molecules that can bind to AMPs and prevent them from reaching the cell membrane. The third stage is the peptide interaction with the lipids in the cell membrane. This interaction can be influenced by flippases and floppases, enzymes that remodel the cell membrane constantly and transform the lipid composition (139–142). Finally, if AMPs do reach the membrane or intracellular space, they can be expelled by endogenous transporters (143–145).

While a few mechanisms for evasion of AMPs activity have been documented in Mtb, three mechanisms of resistance to AMPs are recognized in Gram-Positive bacilli: modifications in membrane/cell wall structure, transport systems and efflux pumps, and AMP-induced gene expression/repression (146).

Changing the composition of the membrane is a strategy used by many bacteria to survive the action of AMPs. This strategy has been reported in several *species of Bacillus*, *L. monocytogenes*, *C. difficile, C. butyricum*, *Lactobacillus plantarum*, *Lactobacillus reuteri and Listeria innocua* (146). Among those changes, the insertion of D-alanine in the lipoteichoic acids (D-alanylation) is used to reduce the negative membrane charge, thus inhibiting interaction with AMPs. Another mechanism of envelope modification is lysinylation of the membrane, meaning the addition of L-lysine to the PG through the action of the protein MprF. Bacteria can modify cell wall components such as the peptidoglycan and O-acetylation of the peptidoglycan can reduce killing by lysozyme. Modifications on the cell wall also include N-deacetylation of the peptidoglycan and glycosylation of the wall teichoic acids (indispensable for protection against the lysozyme, LL-37, and CRAMP). The deacetylation of the N-acetylmuramic acid also confers resistance against lysozyme attack. Changes in lipid composition can interfere with AMPs action. For example, different proportions of lipids are found in bacteriocin-resistant strains. Changes in the proton motive force, *via* FoF1 ATPase, altered the membrane potential related to nisin resistance. Irregular, and more cationic membranes compared to the wild type, have been attributed to mutations in the *pgsA* gene. These mechanisms have been reviewed elsewhere (146).

To avoid antimicrobial host defense, microbes can expel molecules using efflux pumps or ABC transporters. The same mechanism has been implicated in the expulsion of AMPs (147–149). Examples are documented in *B. subtilis*, *B. licheniformis*, *C. difficile*, and *L. monocytogenes* (146).

Cell wall signaling can trigger the expression of many resistance-related genes, such as sigma (s) factors and global regulators in bacteria. Such mechanisms have been reported for *L. monocytogenes*, *B. subtilis, Clostridium difficile*, *Lactococcus lactis* and *Lactococcus garvieae, L. plantarum*, *Leuconostoc mesenteroides*, *Lactobacillus salivarius*, *Lactobacillus acidophilus and Listeria innocua* (146).

Coming back to Mtb, the assembly of its cell envelope is the primary target of anti-tubercular agents (78). Microbes can also develop resistance to AMPs during evolution without added fitness cost and AMP resistant strains may even have competitive advantage over wild-type strains (150). Therefore, multiple regulatory mechanisms, including post-translational modifications (PTMs), are a subject of study in Mtb. Acylation (modification of proteins *via* the attachment of functional groups through acyl linkages) and glycosylation are PTMs that are often found alongside each other. Acylation is important for the localization of proteins in the mycobacterial cell wall, as well as a major contributor to host-pathogen interactions. The mechanism and function of protein glycosylation are only beginning to be understood (151, 152). The glycoproteomic patterns of clinical isolates of the Mtb complex (MTBC) representing different lineages were recently characterized. O-glycosylation constituted 83% of the events identified, while 17% of the sites were N-glycosylated. The main groups of Mtb glycoproteins were involved in cell envelope biosynthesis, fatty acid and lipid metabolism, two-component systems, and pathogen-host interaction (surface exposed or located in the cell wall). The authors conclude that the differential glycosylation pattern may contribute to phenotypic variabilities across Mtb lineages (153). Additionally, lipoglycosylated proteins play a role in lipid transport, including virulence factors such as PDIM and LAM. The full impact of protein glycosylation on the physiology of mycobacteria and immunity to Mtb as well as the impact on glycosylation of the function of individual proteins requires further research (151). Mechanisms such as lysine acetylation are also involved in Mtb virulence and pathogenesis (154, 155), with the Nε- and O-acetylation of Mtb reported involved in central metabolism, translation, stress responses, and antimicrobial drug resistance (156).

The expression of proteins responsible for AMP resistance in Gram positive-bacilli have also been documented in Mtb, such as ABC transporters (157, 158) or mycobacterial respiratory complexes (a promising new drug target for combatting drug-resistant strains of Mtb) (159, 160). However, to our knowledge, the long list of evasion mechanisms described for other Gram-positive bacilli has not been studied in Mtb, with the exception for lysinylation of phosphatidylglycerol (L-PG). It should not be ruled out, though, that Mtb could bear all or some of those mechanisms. Even under evolutive pressure, Mtb could be capable of developing new mechanisms to avoid AMP activity.

The bacterial cell membrane accomplishes controlled exchange of molecules with the extracellular space and mediates specific interactions with the environment. However, the cytoplasmic membrane also includes vulnerable targets for antimicrobial agents. A common feature of cationic antimicrobial peptides (CAMPs) produced by other bacteria or by the host immune system is to utilize the negative charge of bacterial phospholipids such as phosphatidylglycerol (PG) or cardiolipin (CL) for initial adherence and subsequent penetration into the membrane bilayer. To resist CAMPs, many bacteria integrate positive charges into the membrane surface. This is achieved by aminoacylation of negatively charged (PG) or (CL) with alanine, arginine, or lysine residues (161). As a large family of membrane proteins crucial for bacterial physiology and virulence, the multiple peptide resistance factors (MprFs) utilize two separate domains to synthesize and translocate aminoacyl phospholipids to the outer leaflets of bacterial membranes by mechanisms recently reported (162). The two-domain lysyl-transferase (mprF)-lysyl-tRNA synthetase (lysU) protein encoded by the *lysX* gene is used to produce lysinylated phosphatidylglycerol (L-PG) in Mtb, one of the basic phospholipids (PLs) of its membrane (123). These enzymes constitute an important defense mechanism for Mtb as the introduction of positive charges onto the cytoplasmic membrane generates reduced affinity towards cationic AMPs and increases resistance to acidic environments. The mutant Mtb *lysX* showed altered membrane potential and increased sensitivity to cationic antibiotics and peptides. The *lysX* mutant increased the production of pro-inflammatory cytokines in infected macrophages and showed growth defects in the lungs of mice and guinea pigs, thus indicating the important role of *lysX* function for complete virulence (123). Expression of *lysX* was associated with the virulence of Mtb strains (163). Studying a group of well-characterized Mtb clinical isolates, *lysX* mutated strains, and reference strains, the authors showed a significant increase of *lysX* expression in the presence of AMPs. Those strains with higher expression of *lysX* also had increased levels of intracellular survival *in vivo* and *in vitro*, which induced more severe pneumonic lesion in an experimental model of pulmonary TB. The ability of Mtb to replicate intracellularly is directly correlated with the level of *lysX* expressed. Therefore, the amount of *lysX* produced by bacilli modulates the virulence of Mtb (163).

Recently, Boldrin and colleagues (164) reported that *lysX2* is a prototype of a new class within the MprF-like protein family that likely enhances the survival of pathogenic species through its catalytic domain, which is exposed to the extra cytoplasmic side of the cell membrane and is required to decrease the negative charge on the bacterial surface. LysX2 expression in *M. smegmatis* increased cell resistance to HBD-2 and sodium nitrite, improved cell viability, and delayed biofilm formation in an acidic pH environment. *lysX2* significantly reduced the negative charge on the bacterial surface upon exposure to an acidic environment. The authors reported *lysX2* orthologues in major human pathogens and in rapidly growing mycobacteria frequently associated with human infections, but not in environmental and nonpathogenic mycobacteria. Although the Mtb proteome presents a protein with 100% identity compared to the reported *lysX2* prototype, it has not been reported whether it has the same functions as *lysX* (164).

Thanks to their diverse mechanism of action, the main advantage of using AMPs compared to antibiotics is their broad-spectrum activity and their potential to synergize with the conventional antibiotics against TB. AMPs act against TB bacteria disrupting mycobacterial membrane and forming pores, therefore having the ability to target microbial pathogens within eukaryotic cells (165).

## Can AMPs and anti-tuberculous drugs have a potential synergy?

The first works to explore the strategy of combining AMPs with standard antibiotics to improve TB treatment were reported in 2004. Fattorini et al. studied the activity of two AMPs, protegrin-1 (PG-1) and HBD-1, both alone and in combination with isoniazid (INH). Performing *in vitro* assays, they show that PG-1 and HBD-1 alone inhibited mycobacterial growth in drug-resistant and in MDR strains. The combination of AMP + INH enhanced the reduction in mycobacterial growth compared to AMP or INH alone (33). Other researchers evaluated the combination of HNP-1 with INH or RIF, showing a decrease in the MIC of these drugs against *Mtb* H37Rv. The synergism may be explained by an enhanced permeation of the cell membrane (50).

More recently, the activity of a family of six AMPs containing all-D amino acids (D-LAK peptides) was evaluated both *in vitro* and in THP-1 cells (macrophage model) against MDR and XDR clinical strains of Mtb. Their results showed that all D-LAK peptides successfully inhibited Mtb growth *in vitro* and were similarly effective against MDR and XDR strains. D-LAK peptides break the clumping of mycobacteria in broth culture (detergent-like effect), preventing bacteria cell aggregation. The D-LAK120-A peptide was effective as an adjunct agent at nontoxic concentration to enhance the efficacy of INH against drug-resistant Mtb *in vitro*, possibly by facilitation of INH access to mycobacteria by increasing the surface permeability of the pathogen (70). Another study demonstrated the importance of the membrane lytic mechanism for improving anti-TB action of antibiotics. The researchers found that M(LLKK)2M peptides show synergism with RIF against different strains. The peptide M(LLKK)2M was bactericidal and destroyed mycobacteria *via* a membrane-lytic mechanism in experiments visualized by confocal microscopy. Mycobacteria did not acquire resistance with repeated exposures to sublethal doses of the peptide; therefore, the combination treatment would be potentially beneficial to delay anti-TB antibiotic resistance (71).

Bacteriocin AS-48 is an antibacterial peptide produced by *Enterococcus faecalis* and is active against several Gram-positive bacteria. Aguilar-Perez et al. (166), have found that AS-48 was active against Mtb, including H37Rv and other reference and clinical strains. The combination of AS-48 with ethambutol increased the antituberculosis action of AS-48, showing a synergistic interaction. AS-48 exhibits a MIC close to some MICs of the first-line antituberculosis agents. The inhibitory activity of AS-48 and its synergistic combination with ethambutol were also observed on Mtb-infected macrophages. AS-48 did not show any cytotoxicity against macrophage cell lines THP-1, MHS, and J774.2 at concentrations close to its MIC (166). Combination therapy of synthetic HHC-8 antimicrobial peptides (KIWWWWRKR), or MM-10 antimicrobial peptides loaded in poly-ε-caprolactone nanoparticles, displayed synergy against mycobacteria with RIF (167). Their findings suggest that enhanced efficacy is due to protection offered by AMPs encapsulation, resulting in increased membrane permeation by AMPs and increased accumulation of antibiotics within mycobacteria. Future *in vivo* studies would contribute to the development of additional potential drugs for antituberculosis therapy. The fight against TB obligates us to keep searching for new alternatives to current treatments. Because of their nonspecific mode of action, synthetic and natural AMPs have a promising future as wide-spectrum antimicrobials.

## Where the research is going regarding the interaction of AMPs with Mtb cell wall

Since AMPs act through different modalities, extensive efforts have explored their potential as new therapeutic agents against infectious diseases, with some of these molecules already being studied in current clinical trials (130–135). AMPs approved for use in clinical settings can be consulted extensively in a recent review (132). However, most AMPs still face major challenges before arriving to clinical application, the main one being their susceptibility to proteolytic enzymes. Oral administration of AMPs will be affected by enzymes such as pepsin, trypsin, and chymotrypsin. Intravenous administration has to deal with many proteases in the blood (168). Furthermore, intravenous administration leads to shorter half-life due to liver and renal clearances (169). New design strategies have attempted to overcome these challenges (134).

In the design or redesign of AMPs, the ability of bacteria to develop resistance mechanisms to evade them must be considered. The killing ability against bacteria can be achieved by modifying AMPs through strategies such as structural change, amino acid substitution, conjugation with cell-penetration peptides (CPPs), terminal acetylation and amidation, modifying AMPs with organometallic agents and encapsulation with nanoparticles in order to improve the antimicrobial efficacy, reduce toxicity, and accomplish local delivery of AMPs, as reviewed in (135, 170, 171).

Today, *in silico* tools (GenBank, RefSeq, TPA, SwissProt, PDB, NCBI, Protein BLAST, UniProt, InterPro, RCSB PDB, SWORD, EMBL, DDBJ, TrEMBL, etc) are useful for searching for natural AMPs in the genome, proteome, and transcriptome (172–174). A peptide derived from a signal peptide sequence was turned into an AMP through the Joker algorithm. Evaluation of its antimicrobial activity with the microdilution method, and of the membrane integrity using fluorescent probes and scanning electron microscopy imaging, allowed the authors conclude that the modified peptide can kill bacteria by acting on bacterial membranes (175). The transformation of an inactive signal peptide into an AMP is thus a novel approach for creating AMPs. *In silico* design of the AMP motifs have been possible thanks to the antimicrobial peptide databases (APDs), as well as to online tools for AMP screening and identification (176). Examples of successful results are pleurocidin, an AMP found in fish, which displayed *in silico* antimicrobial potential (177). Also, DP7 designed *in silico* showed broad-spectrum antimicrobial activity against MDR bacteria (178).

Although some AMPs show the ability to kill pathogenic bacteria *in vitro and in vivo* in physiological environments with high salt concentration, pH change, and enzyme cleavage, their antimicrobial activity is not as good (179). For this reason, modification and optimization of AMPs are important areas of research.

Amino acid substitution is a strategy to improve the killing activity of AMPs, which includes the substitution of natural L-amino acids with D or unnatural amino acids. For example, the peptide UP09, derived from cationic AMP Pep05, increased its antimicrobial activity against *P. aeruginosa* and showed lower cytotoxicity to host cells *in vivo* when modified to substitute N- and C-terminal amino acids with unnatural amino acids (180). Charge and hydrophobicity are critically important for cationic AMPs activity. In searching the database of antimicrobial activity and structure of peptides (DBAASP), an abundance of bulky hydrophobic and/or aromatic amino acids (Phe, Ile, Leu, Trp and His) have been reported as characteristics of linear AMPs, while Cys, Lys and Gly are rich in cyclic and disulfide-bonded peptides, and Pro, Ser, and Thr increase in cyclic peptides (181). Furthermore, unnatural amino acid residues such as 4-aminobutanoic acid and azulenyl-alanine, have been applied to AMP to improve their killing efficacy and proteolytic resistance (180, 182, 183).

Oligo-N- substituted glycines, also known as antimicrobial peptoids (184), are sequence-specific synthetic peptidomimetics with a peptide backbone, different from AMP in that the side chains are attached to the amide nitrogen of the backbone instead of the alpha carbon (185, 186). This structural difference implies that unknown proteases will recognize and degrade the peptoid structure.

The development of smart chimeric peptides (SCPs) is another strategy to improve the antimicrobial activity of AMPs. A research group designed SCP by connecting LPS-binding peptide (LBP)14 with a marine AMP-N6, showing that the new molecule exhibited increased killing activity against MDR *E. coli*, while it can neutralize LPS (187). Conjugation of CPPs to AMPs can also improve their bactericidal activity.

The structural modification of AMPs can improve their activity and stability. The attachment of AMPs to a helical structure increases their resistance to protease by hiding the proteolytic targets (188). An α-helical structure may also increase the antimicrobial activity of AMPs, as demonstrated with a melittin-relative peptide (AR-23) (189), and decrease their cytotoxicity, as reported for the antifungal peptide Cm-p5 (184).

Three works on the study of anti-TB activity of synthetic peptides identified a relationship between the amino acid sequences of the peptides and their antimicrobial activity against Mtb, suggesting that the increase in tryptophan and lysine content enhances the antimicrobial properties while the increase in alanine, isoleucine, leucine, and valine presence suppresses activity (30, 72, 190). However, contradictory reports have shown that HNP-1, with its four native alanine residues, exhibited greater activity than HNP-2, a molecule with three alanine amino acids (91). It therefore remains a challenge to accurately define the relationship between peptide sequences and their anti-TB activity. Some peptides such as E50-52 have shown anti-mycobacterial activity at not cytotoxic concentrations (106), but for other peptides this is still a concern. Regarding the known property of unnatural and cyclic amino acids and cyclic peptides to increase the *in vivo* and oral bioavailability of AMPs, ecumicin was found with good plasma stability (97). Peptides such as nisin A, lacticin 3147, rufomycin, lassomycin, wollamides, trichoderins, peptoids and BB-3497 may also have good stability. Plasma stability could potentially be improved by introducing unnatural aminoacids and cyclic peptides into their structures.

The search for solutions to deliver AMPs at specific sites has been and continues to be intensive (135, 170, 191–193). Drug delivery systems, using vehicles such as nanoparticles, liposomes or different gel formulations, have been a strategy used to reduce proteolytic degradation (194). Thanks to nanoparticles, the protection of peptides, controlled plasma levels, prolonged and/or controlled release, and reduced administration frequency are becoming possible. These advantages imply lower toxicity to the host (195), and large-scale synthesis of AMP and biotechnological tools continues to improve (196, 197).

Nanotechnology provides strategies for the delivery of AMPs, promoting their stability, toxicity, and target selectivity (198). Some forms of nanoparticles to deliver AMPs are Lipid-Based Nanoparticles, Metal-Based Nanoparticles and Self-Assembling Nanoparticles. To illustrate, liposomes modified with antimicrobial peptides (WLBU2) have shown strong antimicrobial activity against MRSA and *P. aeruginosa* (199). Silver nanoparticles (AgNPs) have shown antimicrobial activity against bacteria (200, 201). The combination of AgNP and the Tet-213 peptide KRWWK- WWRRC exhibits synergistic bactericidal activity (202). AMP-conjugated AuNPs display increased antimicrobial activity and stability in serum and low cytotoxicity in human cells (203). Self-assembling peptide nanomaterials exhibit low toxicity and resistance to high salt conditions, as well as protease degradation. Furthermore, they are injectable and biocompatible (135, 204). A recent report evaluated magnesium oxide and zinc oxide nanoparticles against MDR Mtb, finding bactericide behavior and a possible synergistic effect (205).

## Conclusion

Compared to already existing anti-TB regimens, AMPs have several advantages. Firstly, AMPs have a broad-spectrum activity against different strains of *M. tuberculosis*, including drug-resistant strains. This makes them potential candidates for the treatment of drug-resistant TB, which is a significant public health challenge. Secondly, AMPs have a rapid mode of action, which means that they can kill *M. tuberculosis* quickly, reducing the duration of treatment. Thirdly, AMPs have a low risk of inducing drug resistance, as they target multiple bacterial cell components simultaneously, making it difficult for the bacteria to develop resistance. Finally, AMPs have a good safety profile, as they are part of the human innate immune system and are well-tolerated by the body. These advantages make AMPs an attractive option for the development of new anti-TB therapies, and they hold promise for addressing the challenges associated with the current TB treatment regimens.

There is still a lot of work that needs to be done to better understand the activity of AMPs versus Mtb. We must not forget that even though AMPs are very good candidates, we still need to examine other characteristics they possess, such as their ability to induce angiogenesis and immunomodulation that may lead to collateral undesired effects. However, the scientific community should not remove the spotlight from these promising peptides.

## Author contributions

YJ-D: Writing, reviewing, and editing. AR-C: Writing, reviewing, and editing. CS: Writing, reviewing, and editing. BR-S: Conceptualization, supervision, writing, reviewing, and editing. All authors contributed to the article and approved the submitted version.

## References

[1] Brennan PJ. Structure, function, and biogenesis of the cell wall of mycobacterium tuberculosis. Tuberculosis (Edinb) (2003) 83(1-3):91–7. doi: 10.1016/S1472-9792(02)00089-6 12758196

[2] Dulberger CL Rubin EJ Boutte CC. The mycobacterial cell envelope - a moving target. Nat Rev Microbiol (2020) 18(1):47–59. doi: 10.1038/s41579-019-0273-7 31728063

[3] Koh KW Lehming N Seah GT. Degradation-resistant protein domains limit host cell processing and immune detection of mycobacteria. Mol Immunol (2009) 46(7):1312–8. doi: 10.1016/j.molimm.2008.11.008 19128836

[4] Saini NK Baena A Ng TW Venkataswamy MM Kennedy SC Kunnath-Velayudhan S. Suppression of autophagy and antigen presentation by mycobacterium tuberculosis PE_PGRS47. Nat Microbiol (2016) 1(9):16133. doi: 10.1038/nmicrobiol.2016.133 27562263 PMC5662936

[5] Silva S Vale N. Cationic antimicrobial peptides for tuberculosis: a mini-review. Curr Protein Pept Sci (2019) 20(9):885–92. doi: 10.2174/1389203720666190626160057 31241433

[6] Kathirvel M Mahadevan S. The role of epigenetics in tuberculosis infection. Epigenomics (2016) 8(4):537–49. doi: 10.2217/epi.16.1 27035266

[7] Khadela A Chavda VP Postwala H Shah Y Mistry P. Epigenetics in tuberculosis: immunomodulation of host immune response. Vaccines (Basel) (2022) 10(10):1740. doi: 10.3390/vaccines10101740 36298605 PMC9611989

[8] WHO. Global tuberculosis report (2021). Available at: https://www.who.int/health-topics/tuberculosis#tab=tab_1.

[9] WHO. Global tuberculosis report (2022). Available at: https://www.who.int/teams/global-tuberculosis-programme/tb-reports/global-tuberculosis-report-2022/covid-19-and-tb.

[10] Cambier CJ Falkow S Ramakrishnan L. Host evasion and exploitation schemes of mycobacterium tuberculosis. Cell (2014) 159(7):1497–509. doi: 10.1016/j.cell.2014.11.024 25525872

[11] Zumla A Raviglione M Hafner R von Reyn CF. Tuberculosis. N Engl J Med (2013) 368(8):745–55. doi: 10.1056/NEJMra1200894 23425167

[12] Verrall AJ Netea MG Alisjahbana B Hill PC van Crevel R. Early clearance of mycobacterium tuberculosis: a new frontier in prevention. Immunology (2014) 141(4):506–13. doi: 10.1111/imm.12223 PMC3956425 24754048

[13] Chiaradia L Lefebvre C Parra J Marcoux J Burlet-Schiltz O Etienne G. Dissecting the mycobacterial cell envelope and defining the composition of the native mycomembrane. Sci Rep (2017) 7(1):12807. doi: 10.1038/s41598-017-12718-4 28993692 PMC5634507

[14] Stokas H Rhodes HL Purdy GE. Modulation of the m. tuberculosis cell envelope between replicating and non-replicating persistent bacteria. Tuberculosis (Edinb) (2020) 125:102007. doi: 10.1016/j.tube.2020.102007 33035766 PMC7704923

[15] Brennan P Nikaido H. The envelope of mycobacteria. Annu Rev Biochem (1995) 64:29–63. doi: 10.1146/annurev.bi.64.070195.000333 7574484

[16] Rastogi N. Structure and functions of the cell envelope in relation to mycobacterial virulence, pathogenicity and multiple drug resistance. Res Microbiol (1991) 142:419. doi: 10.1016/0923-2508(91)90112-N 1678545

[17] Grzegorzewicz AE. Assembling of the mycobacterium tuberculosis cell wall core. J Biol Chem (2016) 291(36):18867–79. doi: 10.1074/jbc.M116.739227 PMC5009262 27417139

[18] Singh P Rameshwaram NR Ghosh S Mukhopadhyay S. Cell envelope lipids in the pathophysiology of mycobacterium tuberculosis. Future Microbiol (2018) 13:689–710. doi: 10.2217/fmb-2017-0135 29771143

[19] Ghazaei C. Mycobacterium tuberculosis and lipids: insights into molecular mechanisms from persistence to virulence. J Res Med Sci (2018) 23:63. doi: 10.4103/jrms.JRMS_904_17 30181745 PMC6091133

[20] Maitra A Munshi T Healy J Martin LT Vollmer W Keep NH. Cell wall peptidoglycan in mycobacterium tuberculosis: an achilles’ heel for the TB-causing pathogen. FEMS Microbiol Rev (2019) 43(5):548–75. doi: 10.1093/femsre/fuz016 PMC6736417 31183501

[21] Justen AM Hodges HL Kim LM Sadecki PW Porfirio S Ultee E. Polysaccharide length affects mycobacterial cell shape and antibiotic susceptibility. Sci Adv (2020) 6(38):eaba4015. doi: 10.1126/sciadv.aba4015 32938674 PMC7494350

[22] Singh G Kumar A Maan P Kaur J. Cell wall associated factors of mycobacterium tuberculosis as major virulence determinants: current perspectives in drugs discovery and design. Curr Drug Targets (2017) 18(16):1904–18. doi: 10.2174/1389450118666170711150034 28699515

[23] Makinoshima H Glickman MS. Regulation of mycobacterium tuberculosis cell envelope composition and virulence by intramembrane proteolysis. Nature (2005) 436(7049):406–9. doi: 10.1038/nature03713 PMC1502149 16034419

[24] Phoenix D Dennison S Harris F. Antimicrobial peptides: their history, evolution, and functional promiscuity. Antimicrobial Peptides (2013) p:1–37. doi: 10.1002/9783527652853.ch1

[25] Boparai JK Sharma PK. Mini review on antimicrobial peptides, sources, mechanism and recent applications. Protein Pept Lett (2020) 27(1):4–16. doi: 10.2174/18755305MTAwENDE80 31438824 PMC6978648

[26] Valdez-Miramontes CE De Haro-Acosta J Aréchiga-Flores CF Verdiguel-Fernández L Rivas-Santiago B. Antimicrobial peptides in domestic animals and their applications in veterinary medicine. Peptides (2021) 142:170576. doi: 10.1016/j.peptides.2021.170576 34033877

[27] Rivas-Santiago B Jacobo-Delgado Y Rodriguez-Carlos A. Are host defense peptides and their derivatives ready to be part of the treatment of the next coronavirus pandemic? Arch Immunol Ther Exp (Warsz) (2021) 69(1):25. doi: 10.1007/s00005-021-00630-9 34529143 PMC8444179

[28] Huan Y Kong Q Mou H Yi H. Antimicrobial peptides: classification, design, application and research progress in multiple fields. Front Microbiol (2020) 11:582779. doi: 10.3389/fmicb.2020.582779 33178164 PMC7596191

[29] Stenger S Hanson DA Teitelbaum R Dewan P Niazi KR Froelich CJ. An antimicrobial activity of cytolytic T cells mediated by granulysin. Science (1998) 282(5386):121–5. doi: 10.1126/science.282.5386.121 9756476

[30] Miyakawa Y Ratnakar P Rao AG Costello ML Mathieu-Costello O Lehrer RI. In vitro activity of the antimicrobial peptides human and rabbit defensins and porcine leukocyte protegrin against mycobacterium tuberculosis. Infect Immun (1996) 64(3):926–32. doi: 10.1128/iai.64.3.926-932.1996 PMC173858 8641802

[31] Sharma S Verma I Khuller GK Eur Respir J. Antibacterial activity of human neutrophil peptide-1 against mycobacterium tuberculosis H37Rv: in vitro and ex vivo study. Eur Respir J (2000) 16(1):112–7. doi: 10.1034/j.1399-3003.2000.16a20.x 10933095

[32] Tan BH Meinken C Bastian M Bruns H Legaspi A Ochoa MT. Macrophages acquire neutrophil granules for antimicrobial activity against intracellular pathogens. J Immunol (2006) 177(3):1864–71. doi: 10.4049/jimmunol.177.3.1864 16849498

[33] Fattorini L Gennaro R Zanetti M Tan D Brunori L Giannoni F. In vitro activity of protegrin-1 and beta-defensin-1, alone and in combination with isoniazid, against mycobacterium tuberculosis. Peptides (2004) 25(7):1075–7. doi: 10.1016/j.peptides.2004.04.003 15245864

[34] Rivas-Santiago B Rivas Santiago CE Castañeda-Delgado JE León-Contreras JC Hancock RE Hernandez-Pando R. Activity of LL-37, CRAMP and antimicrobial peptide-derived compounds E2, E6 and CP26 against mycobacterium tuberculosis. Int J Antimicrobial Agents (2013) 41(2):143–8. doi: 10.1016/j.ijantimicag.2012.09.015 23141114

[35] Kisich KO Heifets L Higgins M Diamond G. Antimycobacterial agent based on mRNA encoding human beta-defensin 2 enables primary macrophages to restrict growth of mycobacterium tuberculosis. Infect Immun (2001) 69(4):2692–9. doi: 10.1128/IAI.69.4.2692-2699.2001 PMC98208 11254636

[36] Rivas-Santiago B Schwander SK Sarabia C Diamond G Klein-Patel ME Hernandez-Pando R. Human β-defensin 2 is expressed and associated with mycobacterium tuberculosis during infection of human alveolar epithelial cells. Infect Immun (2005) 73(8):4505–11. doi: 10.1128/IAI.73.8.4505-4511.2005 PMC1201238 16040961

[37] Gutsmann T. Interaction between antimicrobial peptides and mycobacteria. Biochim Biophys Acta (BBA) - Biomembranes (2016) 1858(5):1034–43. doi: 10.1016/j.bbamem.2016.01.031 26851776

[38] Rivas-Santiago B Sada E Tsutsumi V Aguilar-Leon D Contreras JL Hernandez-Pando R. Beta-defensin gene expression during the course of experimental tuberculosis infection. J Infect Dis (2006) 194(5):697–701. doi: 10.1086/506454 16897670

[39] Castaneda-Delgado J Hernández-Pando R Serrano CJ Aguilar-León D León-Contreras J Rivas-Santiago C. Kinetics and cellular sources of cathelicidin during the course of experimental latent tuberculous infection and progressive pulmonary tuberculosis. Clin Exp Immunol (2010) 161(3):542–50. doi: 10.1111/j.1365-2249.2010.04199.x PMC2962973 20636399

[40] Rivas-Santiago B Hernandez-Pando R Carranza C Juarez E Contreras JL Aguilar-Leon D. Expression of cathelicidin LL-37 during mycobacterium tuberculosis infection in human alveolar macrophages, monocytes, neutrophils, and epithelial cells. Infect Immun (2008) 76(3):935–41. doi: 10.1128/IAI.01218-07 PMC2258801 18160480

[41] Gupta S Winglee K Gallo R Bishai WR. Bacterial subversion of cAMP signalling inhibits cathelicidin expression, which is required for innate resistance to mycobacterium tuberculosis. J Pathol (2017) 242(1):52–61. doi: 10.1002/path.4878 28097645 PMC5397332

[42] Wimley WC. Describing the mechanism of antimicrobial peptide action with the interfacial activity model. ACS Chem Biol (2010) 5(10):905–17. doi: 10.1021/cb1001558 PMC2955829 20698568

[43] Jiang Z Higgins MP Whitehurst J Kisich KO Voskuil MI Hodges RS. Anti-tuberculosis activity of α-helical antimicrobial peptides: de novo designed l- and d-enantiomers versus l- and d-LL-37. Protein Pept Lett (2011) 18(3):241–52. doi: 10.2174/092986611794578288 PMC3263701 20858205

[44] Sonawane A Santos JC Mishra BB Jena P Progida C Sorensen OE. Cathelicidin is involved in the intracellular killing of mycobacteria in macrophages. Cell Microbiol (2011) 13(10):1601–17. doi: 10.1111/j.1462-5822.2011.01644.x 21790937

[45] Lourenzoni MR Namba AM Caseli L Degrève L Zaniquelli ME. Study of the interaction of human defensins with cell membrane models: relationships between structure and biological activity. J Phys Chem B (2007) 111(38):11318–29. doi: 10.1021/jp067127g 17784741

[46] Rivas-Santiago CE Rivas-Santiago B León DA Castañeda-Delgado J Hernández Pando R. Induction of β-defensins by l-isoleucine as novel immunotherapy in experimental murine tuberculosis. Clin Exp Immunol (2011) 164(1):80–9. doi: 10.1111/j.1365-2249.2010.04313.x PMC3074220 21235540

[47] Pulido D Torrent M Andreu D Nogués MV Boix E. Two human host defense ribonucleases against mycobacteria, the eosinophil cationic protein (RNase 3) and RNase 7. Antimicrob Agents Chemother (2013) 57(8):3797–805. doi: 10.1128/AAC.00428-13 PMC3719706 23716047

[48] Torres-Juarez F Touqui L Leon-Contreras J Rivas-Santiago C Enciso-Moreno JA Hernández-Pando R. RNase 7 but not psoriasin nor sPLA2-IIA associates with mycobacterium tuberculosis during airway epithelial cell infection. Pathog Dis (2018) 76(2). doi: 10.1093/femspd/fty005 29346642

[49] Sow FB Touqui L Leon-Contreras J Rivas-Santiago C Enciso-Moreno JA Hernández-Pando R. Expression and localization of hepcidin in macrophages: a role in host defense against tuberculosis. J Leukoc Biol (2007) 82(4):934–45. doi: 10.1189/jlb.0407216 17609338

[50] Kalita A Verma I Khuller GK. Role of human neutrophil peptide-1 as a possible adjunct to antituberculosis chemotherapy. J Infect Dis (2004) 190(8):1476–80. doi: 10.1086/424463 15378441

[51] Fu LM. The potential of human neutrophil peptides in tuberculosis therapy. Int J Tuberc Lung Dis (2003) 7(11):1027–32. 14598960

[52] Pazgier M Li X Lu W Lubkowski J. Human defensins: synthesis and structural properties. Curr Pharm Des (2007) 13(30):3096–118. doi: 10.2174/138161207782110381 17979752

[53] Corrales-Garcia L Ortiz E Castañeda-Delgado J Rivas-Santiago B Corzo G. Bacterial expression and antibiotic activities of recombinant variants of human beta-defensins on pathogenic bacteria and m. tuberculosis. Protein Expr Purif (2013) 89(1):33–43. doi: 10.1016/j.pep.2013.02.007 23459290

[54] Deshpande D Grieshober M Wondany F Gerbl F Noschka R Michaelis J. Super-resolution microscopy reveals a direct interaction of intracellular mycobacterium tuberculosis with the antimicrobial peptide LL-37. Int J Mol Sci (2020) 21(18):6741. doi: 10.3390/ijms21186741 32937921 PMC7555347

[55] Foss MH Powers KM Purdy GE. Structural and functional characterization of mycobactericidal ubiquitin-derived peptides in model and bacterial membranes. Biochemistry (2012) 51(49):9922–9. doi: 10.1021/bi301426j PMC3567233 23173767

[56] Alonso S Pethe K Russell DG Purdy GE. Lysosomal killing of mycobacterium mediated by ubiquitin-derived peptides is enhanced by autophagy. Proc Natl Acad Sci U.S.A. (2007) 104(14):6031–6. doi: 10.1073/pnas.0700036104 PMC1851611 17389386

[57] Banerjee DI Gohil TP. Interaction of antimicrobial peptide with mycolyl transferase in mycobacterium tuberculosis. Int J Mycobacteriol (2016) 5(1):83–8. doi: 10.1016/j.ijmyco.2015.07.002 26927995

[58] Schittek B Hipfel R Sauer B Bauer J Kalbacher H Stevanovic S. Dermcidin: a novel human antibiotic peptide secreted by sweat glands. Nat Immunol (2001) 2(12):1133–7. doi: 10.1038/ni732 11694882

[59] Gallo RL Kim KJ Bernfield M Kozak CA Zanetti M Merluzzi L. Identification of CRAMP, a cathelin-related antimicrobial peptide expressed in the embryonic and adult mouse. J Biol Chem (1997) 272(20):13088–93. doi: 10.1074/jbc.272.20.13088 9148921

[60] Yu K Park K Kang SW Shin SY Hahm KS Kim Y. Solution structure of a cathelicidin-derived antimicrobial peptide, CRAMP as determined by NMR spectroscopy. J Pept Res (2002) 60:1–9. doi: 10.1034/j.1399-3011.2002.01968.x 12081622

[61] Portell-Buj E Vergara A Alejo I López-Gavín A Monté MR San Nicolás L. In vitro activity of 12 antimicrobial peptides against mycobacterium tuberculosis and mycobacterium avium clinical isolates. J Med Microbiol (2019) 68(2):211–5. doi: 10.1099/jmm.0.000912 30570475

[62] Ramirez-Carreto S Jiménez-Vargas JM Rivas-Santiago B Corzo G Possani LD Becerril B. Peptides from the scorpion vaejovis punctatus with broad antimicrobial activity. Peptides (2015) 73:51–9. doi: 10.1016/j.peptides.2015.08.014 26352292

[63] Rodriguez A Villegas E Montoya-Rosales A Rivas-Santiago B Corzo G. Characterization of antibacterial and hemolytic activity of synthetic pandinin 2 variants and their inhibition against mycobacterium tuberculosis. PloS One (2014) 9(7):e101742. doi: 10.1371/journal.pone.0101742 25019413 PMC4096598

[64] Abraham P Jose L Maliekal TT Kumar RA Kumar KS. B1CTcu5: a frog-derived brevinin-1 peptide with anti-tuberculosis activity. Peptides (2020) 132:170373. doi: 10.1016/j.peptides.2020.170373 32679168

[65] Ling LL Schneider T Peoples AJ Spoering AL Engels I Conlon BP. A new antibiotic kills pathogens without detectable resistance. Nature (2015) 517(7535):455–9. doi: 10.1038/nature14098 PMC7414797 25561178

[66] Carroll J Draper LA O'Connor PM Coffey A Hill C Ross RP. Comparison of the activities of the lantibiotics nisin and lacticin 3147 against clinically significant mycobacteria. Int J Antimicrob Agents (2010) 36(2):132–6. doi: 10.1016/j.ijantimicag.2010.03.029 20547041

[67] Toro JC Hoffner S Linde C Andersson M Andersson J Grundström S. Enhanced susceptibility of multidrug resistant strains of mycobacterium tuberculosis to granulysin peptides correlates with a reduced fitness phenotype. Microbes Infect (2006) 8(8):1985–93. doi: 10.1016/j.micinf.2006.02.030 16793311

[68] Linde CM Grundström S Nordling E Refai E Brennan PJ Andersson M. Conserved structure and function in the granulysin and NK-lysin peptide family. Infect Immun (2005) 73(10):6332–9. doi: 10.1128/IAI.73.10.6332-6339.2005 PMC1230960 16177304

[69] Andreu D Carreño C Linde C Boman HG Andersson M. Identification of an anti-mycobacterial domain in NK-lysin and granulysin. Biochem J (1999) 344 Pt 3(Pt 3):845–9. doi: 10.1042/bj3440845 PMC1220707 10585872

[70] Lan Y Lam JT Siu GK Yam WC Mason AJ Lam JK. Cationic amphipathic d-enantiomeric antimicrobial peptides with in vitro and ex vivo activity against drug-resistant mycobacterium tuberculosis. Tuberculosis (Edinb) (2014) 94(6):678–89. doi: 10.1016/j.tube.2014.08.001 25154927

[71] Khara JS Wang Y Ke XY Liu S Newton SM Langford PR. Anti-mycobacterial activities of synthetic cationic alpha-helical peptides and their synergism with rifampicin. Biomaterials (2014) 35(6):2032–8. doi: 10.1016/j.biomaterials.2013.11.035 24314557

[72] Ramon-Garcia S Mikut R Ng C Ruden S Volkmer R Reischl M. Targeting mycobacterium tuberculosis and other microbial pathogens using improved synthetic antibacterial peptides. Antimicrob Agents Chemother (2013) 57(5):2295–303. doi: 10.1128/AAC.00175-13 PMC3632949 23478953

[73] Kapoor R Eimerman PR Hardy JW Cirillo JD Contag CH Barron AE. Efficacy of antimicrobial peptoids against mycobacterium tuberculosis. Antimicrob Agents Chemother (2011) 55(6):3058–62. doi: 10.1128/AAC.01667-10 PMC3101442 21464254

[74] Silva JP Gonçalves C Costa C Sousa J Silva-Gomes R Castro AG. Delivery of LLKKK18 loaded into self-assembling hyaluronic acid nanogel for tuberculosis treatment. J Control Release (2016) 235:112–24. doi: 10.1016/j.jconrel.2016.05.064 27261333

[75] Rivas-Santiago B Castañeda-Delgado JE Rivas Santiago CE Waldbrook M González-Curiel I León-Contreras JC. Ability of innate defence regulator peptides IDR-1002, IDR-HH2 and IDR-1018 to protect against mycobacterium tuberculosis infections in animal models. PloS One (2013) 8(3):e59119. doi: 10.1371/journal.pone.0059119 23555622 PMC3605426

[76] Noschka R Wondany F Kizilsavas G Weil T Weidinger G Walther P. Gran1: a granulysin-derived peptide with potent activity against intracellular mycobacterium tuberculosis. Int J Mol Sci (2021) 22(16):8392. doi: 10.3390/ijms22168392 34445098 PMC8395039

[77] Jadhav K Singh R Ray E Singh AK Verma RK. Taming the devil: antimicrobial peptides for safer TB therapeutics. Curr Protein Pept Sci (2022) 23:643–56. doi: 10.2174/1389203723666220526161109 35619262

[78] Mehta K Sharma P Mujawar S Vyas A. Role of antimicrobial peptides in treatment and prevention of mycobacterium tuberculosis: a review. Int J Pept Res Ther (2022) 28(5):132. doi: 10.1007/s10989-022-10435-9 35891800 PMC9305673

[79] Yathursan S Wiles S Read H Sarojini V. A review on anti-tuberculosis peptides: impact of peptide structure on anti-tuberculosis activity. J Pept Sci (2019) 25(11):e3213. doi: 10.1002/psc.3213 31515916

[80] Samuchiwal SK Tousif S Singh DK Kumar A Ghosh A Bhalla K. A peptide fragment from the human COX3 protein disrupts association of mycobacterium tuberculosis virulence proteins ESAT-6 and CFP10, inhibits mycobacterial growth and mounts protective immune response. BMC Infect Dis (2014) 14:355. doi: 10.1186/1471-2334-14-355 24985537 PMC4089558

[81] Sharma S Khuller G. DNA As the intracellular secondary target for antibacterial action of human neutrophil peptide-I against mycobacterium tuberculosis H37Ra. Curr Microbiol (2001) 43(1):74–6. doi: 10.1007/s002840010263 11375668

[82] Pazgier M Hoover DM Yang D Lu W Lubkowski J. Human beta-defensins. Cell Mol Life Sci (2006) 63(11):1294–313. doi: 10.1007/s00018-005-5540-2 PMC11136124 16710608

[83] Nickel D Busch M Mayer D Hagemann B Knoll V Stenger S. Hypoxia triggers the expression of human beta defensin 2 and antimicrobial activity against mycobacterium tuberculosis in human macrophages. J Immunol (2012) 188(8):4001–7. doi: 10.4049/jimmunol.1100976 22427634

[84] Sow FB Nandakumar S Velu V Kellar KL Schlesinger LS Amara RR. Mycobacterium tuberculosis components stimulate production of the antimicrobial peptide hepcidin. Tuberculosis (Edinb) (2011) 91(4):314–21. doi: 10.1016/j.tube.2011.03.003 21482189

[85] Li M Rigby K Lai Y Nair V Peschel A Schittek B. Staphylococcus aureus mutant screen reveals interaction of the human antimicrobial peptide dermcidin with membrane phospholipids. Antimicrob Agents Chemother (2009) 53(10):4200–10. doi: 10.1128/AAC.00428-09 PMC2764178 19596877

[86] Linde CM Hoffner SE Refai E Andersson M. In vitro activity of PR-39, a proline-arginine-rich peptide, against susceptible and multi-drug-resistant mycobacterium tuberculosis. J Antimicrob Chemother (2001) 47(5):575–80. doi: 10.1093/jac/47.5.575 11328767

[87] Pruksakorn P Arai M Kotoku N Vilchèze C Baughn AD Moodley P. Trichoderins, novel aminolipopeptides from a marine sponge-derived trichoderma sp., are active against dormant mycobacteria. Bioorg Med Chem Lett (2010) 20(12):3658–63. doi: 10.1016/j.bmcl.2010.04.100 20483615

[88] Pruksakorn P Arai M Liu L Moodley P Jacobs WR Jr Kobayashi M. Action-mechanism of trichoderin a, an anti-dormant mycobacterial aminolipopeptide from marine sponge-derived trichoderma sp. Biol Pharm Bull (2011) 34(8):1287–90. doi: 10.1248/bpb.34.1287 21804219

[89] Daletos G Kalscheuer R Koliwer-Brandl H Hartmann R de Voogd NJ Wray V. Callyaerins from the marine sponge callyspongia aerizusa: cyclic peptides with antitubercular activity. J Nat Prod (2015) 78(8):1910–25. doi: 10.1021/acs.jnatprod.5b00266 26213786

[90] Ibrahim SR Min CC Teuscher F Ebel R Kakoschke C Lin W. Callyaerins a-f and h, new cytotoxic cyclic peptides from the Indonesian marine sponge callyspongia aerizusa. Bioorg Med Chem (2010) 18(14):4947–56. doi: 10.1016/j.bmc.2010.06.012 20599387

[91] Tenland E Krishnan N Rönnholm A Kalsum S Puthia M Mörgelin M. A novel derivative of the fungal antimicrobial peptide plectasin is active against mycobacterium tuberculosis. Tuberculosis (Edinb) (2018) 113:231–8. doi: 10.1016/j.tube.2018.10.008 PMC6289163 30514507

[92] Gavrish E Sit CS Cao S Kandror O Spoering A Peoples A. Lassomycin, a ribosomally synthesized cyclic peptide, kills mycobacterium tuberculosis by targeting the ATP-dependent protease ClpC1P1P2. Chem Biol (2014) 21(4):509–18. doi: 10.1016/j.chembiol.2014.01.014 PMC4060151 24684906

[93] Rastogi N Labrousse V Goh KS. In vitro activities of fourteen antimicrobial agents against drug susceptible and resistant clinical isolates of mycobacterium tuberculosis and comparative intracellular activities against the virulent H37Rv strain in human macrophages. Curr Microbiol (1996) 33(3):167–75. doi: 10.1007/s002849900095 8672093

[94] Akbergenov R Shcherbakov D Matt T Duscha S Meyer M Wilson DN. Molecular basis for the selectivity of antituberculosis compounds capreomycin and viomycin. Antimicrob Agents Chemother (2011) 55(10):4712–7. doi: 10.1128/AAC.00628-11 PMC3187005 21768509

[95] Shiba T Nomoto S Wakamiya T. The chemical structure of capreomycin. Experientia (1976) 32(9):1109–11. doi: 10.1007/BF01927571 61134

[96] Stanley RE Blaha G Grodzicki RL Strickler MD Steitz TA. The structures of the anti-tuberculosis antibiotics viomycin and capreomycin bound to the 70S ribosome. Nat Struct Mol Biol (2010) 17(3):289–93. doi: 10.1038/nsmb.1755 PMC2917106 20154709

[97] Gao W Kim JY Anderson JR Akopian T Hong S Jin YY. The cyclic peptide ecumicin targeting ClpC1 is active against mycobacterium tuberculosis in vivo. Antimicrob Agents Chemother (2015) 59(2):880–9. doi: 10.1128/AAC.04054-14 PMC4335914 25421483

[98] Gao W Kim JY Chen SN Cho SH Choi J Jaki BU. Discovery and characterization of the tuberculosis drug lead ecumicin. Org Lett (2014) 16(23):6044–7. doi: 10.1021/ol5026603 PMC5450905 25409285

[99] Khalil ZG Salim AA Lacey E Blumenthal A Capon RJ. Wollamides: antimycobacterial cyclic hexapeptides from an Australian soil streptomyces. Org Lett (2014) 16(19):5120–3. doi: 10.1021/ol502472c 25229313

[100] Wolf NM Lee H Choules MP Pauli GF Phansalkar R Anderson JR. High-resolution structure of ClpC1-rufomycin and ligand binding studies provide a framework to design and optimize anti-tuberculosis leads. ACS Infect Dis (2019) 5(6):829–40. doi: 10.1021/acsinfecdis.8b00276 PMC6657506 30990022

[101] Kaku R. [Studies on the antitubercular activity of a new antibiotic, ilamycin. 1. antitubercular activity of water-insoluble ilamycin]. J Antibiot B (1963) 16:93–8. 14042065

[102] Schmitt EK Riwanto M Sambandamurthy V Roggo S Miault C Zwingelstein C. The natural product cyclomarin kills mycobacterium tuberculosis by targeting the ClpC1 subunit of the caseinolytic protease. Angew Chem Int Ed Engl (2011) 50(26):5889–91. doi: 10.1002/anie.201101740 21563281

[103] Vasudevan D Rao SP Noble CG. Structural basis of mycobacterial inhibition by cyclomarin a. J Biol Chem (2013) 288(43):30883–91. doi: 10.1074/jbc.M113.493767 PMC3829403 24022489

[104] Barbie P Kazmaier U. Total synthesis of Cyclomarin A a marine cycloheptapeptide with anti-tuberculosis and anti-malaria activity. Org Lett (2016) 18(2):204–7. doi: 10.1021/acs.orglett.5b03292 26699807

[105] Kiefer A Bader CD Held J Esser A Rybniker J Empting M. Synthesis of new cyclomarin derivatives and their biological evaluation towards mycobacterium tuberculosis and plasmodium falciparum. Chemistry (2019) 25(37):8894–902. doi: 10.1002/chem.201901640 31012978

[106] Sosunov V Mischenko V Eruslanov B Svetoch E Shakina Y Stern N. Antimycobacterial activity of bacteriocins and their complexes with liposomes. J Antimicrob Chemother (2007) 59(5):919–25. doi: 10.1093/jac/dkm053 17347179

[107] Teng T Liu J Wei H. Anti-mycobacterial peptides: from human to phage. Cell Physiol Biochem (2015) 35(2):452–66. doi: 10.1159/000369711 25613372

[108] Yagi A Uchida R Hamamoto H Sekimizu K Kimura KI Tomoda H. Anti-mycobacterium activity of microbial peptides in a silkworm infection model with mycobacterium smegmatis. J Antibiot (Tokyo) (2017) 70(5):685–90. doi: 10.1038/ja.2017.23 28446822

[109] Koyama N Kojima S Nonaka K Masuma R Matsumoto M Omura S. Calpinactam, a new anti-mycobacterial agent, produced by mortierella alpina FKI-4905. J Antibiot (Tokyo) (2010) 63(4):183–6. doi: 10.1038/ja.2010.14 20186169

[110] Nagai K Koyama N Sato N Yanagisawa C Tomoda H. Synthesis and antimycobacterial activity of calpinactam derivatives. Bioorg Med Chem Lett (2012) 22(24):7739–41. doi: 10.1016/j.bmcl.2012.09.069 23116887

[111] Silva JP Appelberg R Gama FM. Antimicrobial peptides as novel anti-tuberculosis therapeutics. Biotechnol Adv (2016) 34(5):924–40. doi: 10.1016/j.biotechadv.2016.05.007 27235189

[112] Sharma A Pohane AA Bansal S Bajaj A Jain V Srivastava A. Cell penetrating synthetic antimicrobial peptides (SAMPs) exhibiting potent and selective killing of mycobacterium by targeting its DNA. Chemistry (2015) 21(9):3540–5. doi: 10.1002/chem.201404650 25608020

[113] Pelaez Coyotl EA Barrios Palacios J Muciño G Moreno-Blas D Costas M Montiel Montes T. Antimicrobial peptide against mycobacterium tuberculosis that activates autophagy is an effective treatment for tuberculosis. Pharmaceutics (2020) 12(11):1071. doi: 10.3390/pharmaceutics12111071 33182483 PMC7697726

[114] Shah P Hsiao FS Ho YH Chen CS. The proteome targets of intracellular targeting antimicrobial peptides. Proteomics (2016) 16(8):1225–37. doi: 10.1002/pmic.201500380 26648572

[115] AlMatar M Makky EA Yakıcı G Var I Kayar B Köksal F. Antimicrobial peptides as an alternative to anti-tuberculosis drugs. Pharmacol Res (2018) 128:288–305. doi: 10.1016/j.phrs.2017.10.011 29079429

[116] Graf M Wilson DN. Intracellular antimicrobial peptides targeting the protein synthesis machinery. Adv Exp Med Biol 2019 (1117) p:73–89. doi: 10.1007/978-981-13-3588-4_6 30980354

[117] Mishra NM Briers Y Lamberigts C Steenackers H Robijns S Landuyt B. Evaluation of the antibacterial and antibiofilm activities of novel CRAMP–vancomycin conjugates with diverse linkers11Electronic supplementary information (ESI) available: detailed experimental procedure, characterization of compounds etc. see DOI: 10.1039/c5ob00830a. Organic Biomolecular Chem (2015) 13(27):7477–86. doi: 10.1039/C5OB00830A 26068402

[118] Cudic M Otvos L Jr. Intracellular targets of antibacterial peptides. Curr Drug Targets (2002) 3(2):101–6. doi: 10.2174/1389450024605445 11958294

[119] Purdy GE Niederweis M Russell DG. Decreased outer membrane permeability protects mycobacteria from killing by ubiquitin-derived peptides. Mol Microbiol (2009) 73(5):844–57. doi: 10.1111/j.1365-2958.2009.06801.x PMC2747030 19682257

[120] Ogata K Linzer BA Zuberi RI Ganz T Lehrer RI Catanzaro A. Activity of defensins from human neutrophilic granulocytes against mycobacterium avium-mycobacterium intracellulare. Infect Immun (1992) 60(11):4720–5. doi: 10.1128/iai.60.11.4720-4725.1992 PMC258223 1398982

[121] Gera JF Lichtenstein A. Human neutrophil peptide defensins induce single strand DNA breaks in target cells. Cell Immunol (1991) 138(1):108–20. doi: 10.1016/0008-8749(91)90136-Y 1913832

[122] Lemaire S Trinh TT Le HT Tang SC Hincke M Wellman-Labadie O. Antimicrobial effects of H4-(86-100), histogranin and related compounds–possible involvement of DNA gyrase. FEBS J (2008) 275(21):5286–97. doi: 10.1111/j.1742-4658.2008.06659.x 18803668

[123] Maloney E Stankowska D Zhang J Fol M Cheng QJ Lun S. The two-domain LysX protein of mycobacterium tuberculosis is required for production of lysinylated phosphatidylglycerol and resistance to cationic antimicrobial peptides. PloS Pathog (2009) 5(7):e1000534. doi: 10.1371/journal.ppat.1000534 19649276 PMC2713425

[124] Rivas-Santiago B Rivas-Santiago C Sada E Hernández-Pando R. Prophylactic potential of defensins and l-isoleucine in tuberculosis household contacts: an experimental model. Immunotherapy (2015) 7(3):207–13. doi: 10.2217/imt.14.119 25804474

[125] Polena H Boudou F Tilleul S Dubois-Colas N Lecointe C Rakotosamimanana N. Mycobacterium tuberculosis exploits the formation of new blood vessels for its dissemination. Sci Rep (2016) 6:33162. doi: 10.1038/srep33162 27616470 PMC5018821

[126] Takahashi M Umehara Y Yue H Trujillo-Paez JV Peng G Nguyen HLT. The antimicrobial peptide human beta-Defensin-3 accelerates wound healing by promoting angiogenesis, cell migration, and proliferation through the FGFR/JAK2/STAT3 signaling pathway. Front Immunol (2021) 12:712781. doi: 10.3389/fimmu.2021.712781 34594328 PMC8476922

[127] Salvado MD Di Gennaro A Lindbom L Agerberth B Haeggström JZ. Cathelicidin LL-37 induces angiogenesis via PGE2-EP3 signaling in endothelial cells, in vivo inhibition by aspirin. Arterioscler Thromb Vasc Biol (2013) 33(8):1965–72. doi: 10.1161/ATVBAHA.113.301851 23766266

[128] Cadena AM Fortune SM Flynn JL. Heterogeneity in tuberculosis. Nat Rev Immunol (2017) 17(11):691–702. doi: 10.1038/nri.2017.69 28736436 PMC6247113

[129] Cronan MR. In the thick of it: formation of the tuberculous granuloma and its effects on host and therapeutic responses. Front Immunol (2022) 13:820134. doi: 10.3389/fimmu.2022.820134 35320930 PMC8934850

[130] Jampilek J Kralova K. Advances in nanostructures for antimicrobial therapy. Materials (Basel) (2022) 15(7):2388. doi: 10.3390/ma15072388 35407720 PMC8999898

[131] Gan BH Gaynord J Rowe SM Deingruber T Spring DR. The multifaceted nature of antimicrobial peptides: current synthetic chemistry approaches and future directions. Chem Soc Rev (2021) 50(13):7820–80. doi: 10.1039/D0CS00729C PMC8689412 34042120

[132] Talapko J Meštrović T Juzbašić M Tomas M Erić S Horvat Aleksijević L. Antimicrobial peptides-mechanisms of action, antimicrobial effects and clinical applications. Antibiotics (Basel) (2022) 11(10):1417. doi: 10.3390/antibiotics11101417 36290075 PMC9598582

[133] Oliveira GS Costa RP Gomes P Gomes MS Silva T Teixeira C. Antimicrobial peptides as potential anti-tubercular leads: a concise review. Pharm (Basel) (2021) 14(4):323. doi: 10.3390/ph14040323 PMC8065624 33918182

[134] Costa F Teixeira C Gomes P Martins MCL. Clinical application of AMPs. Adv Exp Med Biol (2019) 1117:281–98. doi: 10.1007/978-981-13-3588-4_15 30980363

[135] Kang SJ Nam SH Lee BJ. Engineering approaches for the development of antimicrobial peptide-based antibiotics. Antibiotics (Basel) (2022) 11(10):1338. doi: 10.3390/antibiotics11101338 36289996 PMC9599025

[136] Hurdle JG O'Neill AJ Chopra I Lee RE. Targeting bacterial membrane function: an underexploited mechanism for treating persistent infections. Nat Rev Microbiol (2011) 9(1):62–75. doi: 10.1038/nrmicro2474 21164535 PMC3496266

[137] Kmeck A Tancer RJ Ventura CR Wiedman GR. Synergies with and resistance to membrane-active peptides. Antibiotics (Basel) (2020) 9(9):620. doi: 10.3390/antibiotics9090620 32961656 PMC7559582

[138] Nguyen TTH Myrold DD Mueller RS. Distributions of extracellular peptidases across prokaryotic genomes reflect phylogeny and habitat. Front Microbiol (2019) 10:413. doi: 10.3389/fmicb.2019.00413 30891022 PMC6411800

[139] Tannert A Pohl A Pomorski T Herrmann A. Protein-mediated transbilayer movement of lipids in eukaryotes and prokaryotes: the relevance of ABC transporters. Int J Antimicrob Agents (2003) 22(3):177–87. doi: 10.1016/S0924-8579(03)00217-6 13678819

[140] Dawson RJ Locher KP. Structure of a bacterial multidrug ABC transporter. Nature (2006) 443(7108):180–5. doi: 10.1038/nature05155 16943773

[141] Contreras FX Sánchez-Magraner L Alonso A Goñi FM. Transbilayer (flip-flop) lipid motion and lipid scrambling in membranes. FEBS Lett (2010) 584(9):1779–86. doi: 10.1016/j.febslet.2009.12.049 20043909

[142] van der Mark VA Elferink RP Paulusma CC. P4 ATPases: flippases in health and disease. Int J Mol Sci (2013) 14(4):7897–922. doi: 10.3390/ijms14047897 PMC3645723 23579954

[143] Chiang WC Pamp SJ Nilsson M Givskov M Tolker-Nielsen T. The metabolically active subpopulation in pseudomonas aeruginosa biofilms survives exposure to membrane-targeting antimicrobials via distinct molecular mechanisms. FEMS Immunol Med Microbiol (2012) 65(2):245–56. doi: 10.1111/j.1574-695X.2012.00929.x 22251216

[144] Olaitan AO Morand S Rolain JM. Mechanisms of polymyxin resistance: acquired and intrinsic resistance in bacteria. Front Microbiol (2014) 5:643. doi: 10.3389/fmicb.2014.00643 25505462 PMC4244539

[145] El-Sayed Ahmed MAE Zhong LL Shen C Yang Y Doi Y Tian GB. Colistin and its role in the era of antibiotic resistance: an extended review (2000-2019). Emerg Microbes Infect (2020) 9(1):868–85. doi: 10.1080/22221751.2020.1754133 PMC7241451 32284036

[146] Assoni L Milani B Carvalho MR Nepomuceno LN Waz NT Guerra MES. Resistance mechanisms to antimicrobial peptides in gram-positive bacteria. Front Microbiol (2020) 11:593215. doi: 10.3389/fmicb.2020.593215 33193264 PMC7609970

[147] Bernard R Guiseppi A Chippaux M Foglino M Denizot F. Resistance to bacitracin in bacillus subtilis: unexpected requirement of the BceAB ABC transporter in the control of expression of its own structural genes. J Bacteriol (2007) 189(23):8636–42. doi: 10.1128/JB.01132-07 PMC2168949 17905982

[148] Collins B Curtis N Cotter PD Hill C Ross RP. The ABC transporter AnrAB contributes to the innate resistance of listeria monocytogenes to nisin, bacitracin, and various beta-lactam antibiotics. Antimicrob Agents Chemother (2010) 54(10):4416–23. doi: 10.1128/AAC.00503-10 PMC2944581 20643901

[149] McBride SM Sonenshein AL. Identification of a genetic locus responsible for antimicrobial peptide resistance in clostridium difficile. Infect Immun (2011) 79(1):167–76. doi: 10.1128/IAI.00731-10 PMC3019887 20974818

[150] Andersson DI Hughes D Kubicek-Sutherland JZ. Mechanisms and consequences of bacterial resistance to antimicrobial peptides. Drug Resist Update (2016) 26:43–57. doi: 10.1016/j.drup.2016.04.002 27180309

[151] Mehaffy C Belisle JT Dobos KM. Mycobacteria and their sweet proteins: an overview of protein glycosylation and lipoglycosylation in m. tuberculosis. Tuberculosis (Edinb) (2019) 115:1–13. doi: 10.1016/j.tube.2019.01.001 30948163

[152] Tucci P Portela M Chetto CR González-Sapienza G Marín M. Integrative proteomic and glycoproteomic profiling of mycobacterium tuberculosis culture filtrate. PloS One (2020) 15(3):e0221837. doi: 10.1371/journal.pone.0221837 32126063 PMC7053730

[153] Birhanu AG Yimer SA Kalayou S Riaz T Zegeye ED Holm-Hansen C. Ample glycosylation in membrane and cell envelope proteins may explain the phenotypic diversity and virulence in the mycobacterium tuberculosis complex. Sci Rep (2019) 9(1):2927. doi: 10.1038/s41598-019-39654-9 30814666 PMC6393673

[154] Lee HJ Lang PT Fortune SM Sassetti CM Alber T. Cyclic AMP regulation of protein lysine acetylation in mycobacterium tuberculosis. Nat Struct Mol Biol (2012) 19(8):811–8. doi: 10.1038/nsmb.2318 PMC3414669 22773105

[155] Nambi S Basu N Visweswariah SS. cAMP-regulated protein lysine acetylases in mycobacteria. J Biol Chem (2010) 285(32):24313–23. doi: 10.1074/jbc.M110.118398 PMC2915667 20507997

[156] Birhanu AG Yimer SA Holm-Hansen C Norheim G Aseffa A Abebe M. N(epsilon)- and O-acetylation in mycobacterium tuberculosis lineage 7 and lineage 4 strains: proteins involved in bioenergetics, virulence, and antimicrobial resistance are acetylated. J Proteome Res (2017) 16(11):4045–59. doi: 10.1021/acs.jproteome.7b00429 PMC5669810 28920697

[157] Slotboom DJ Ettema TW Nijland M Thangaratnarajah C. Bacterial multi-solute transporters. FEBS Lett (2020) 594(23):3898–907. doi: 10.1002/1873-3468.13912 32810294

[158] de Oliveira CBM Balan A. The ATP-binding cassette (ABC) transport systems in mycobacterium tuberculosis: structure, function, and possible targets for therapeutics. Biol (Basel) (2020) 9(12):443. doi: 10.3390/biology9120443 PMC7761784 33291531

[159] Hards K Cheung CY Waller N Adolph C Keighley L Tee ZS. An amiloride derivative is active against the F(1)F(o)-ATP synthase and cytochrome bd oxidase of mycobacterium tuberculosis. Commun Biol (2022) 5(1):166. doi: 10.1038/s42003-022-03110-8 35210534 PMC8873251

[160] McNeil MB Cheung CY Waller NJE Adolph C Chapman CL Seeto NEJ. Uncovering interactions between mycobacterial respiratory complexes to target drug-resistant mycobacterium tuberculosis. Front Cell Infect Microbiol (2022) 12:980844. doi: 10.3389/fcimb.2022.980844 36093195 PMC9461714

[161] Slavetinsky C Kuhn S Peschel A. Bacterial aminoacyl phospholipids - biosynthesis and role in basic cellular processes and pathogenicity. Biochim Biophys Acta Mol Cell Biol Lipids (2017) 1862(11):1310–8. doi: 10.1016/j.bbalip.2016.11.013 27940309

[162] Song D Jiao H Liu Z. Phospholipid translocation captured in a bifunctional membrane protein MprF. Nat Commun (2021) 12(1):2927. doi: 10.1038/s41467-021-23248-z 34006869 PMC8131360

[163] Montoya-Rosales A Provvedi R Torres-Juarez F Enciso-Moreno JA Hernandez-Pando R Manganelli R. lysX gene is differentially expressed among mycobacterium tuberculosis strains with different levels of virulence. Tuberculosis (Edinb) (2017) 106:106–17. doi: 10.1016/j.tube.2017.07.005 28802397

[164] Boldrin F Cioetto Mazzabò L Lanéelle MA Rindi L Segafreddo G Lemassu A. LysX2 is a mycobacterium tuberculosis membrane protein with an extracytoplasmic MprF-like domain. BMC Microbiol (2022) 22(1):85. doi: 10.1186/s12866-022-02493-2 35365094 PMC8974105

[165] Hollmann A Martinez M Maturana P Semorile LC Maffia PC. Antimicrobial peptides: interaction with model and biological membranes and synergism with chemical antibiotics. Front Chem (2018) 6:204. doi: 10.3389/fchem.2018.00204 29922648 PMC5996110

[166] Aguilar-Perez C Gracia B Rodrigues L Vitoria A Cebrián R Deboosère N. Synergy between circular bacteriocin AS-48 and ethambutol against mycobacterium tuberculosis. Antimicrob Agents Chemother (2018) 62(9):e00359-18. doi: 10.1128/AAC.00359-18 29987141 PMC6125546

[167] Sharma A Gaur A Kumar V Sharma N Patil SA Verma RK. Antimicrobial activity of synthetic antimicrobial peptides loaded in poly-e-caprolactone nanoparticles against mycobacteria and their functional synergy with rifampicin. Int J Pharm (2021) 608:121097. doi: 10.1016/j.ijpharm.2021.121097 34534632

[168] Haney EF Hancock RE. Peptide design for antimicrobial and immunomodulatory applications. Biopolymers (2013) 100(6):572–83. doi: 10.1002/bip.22250 PMC3932157 23553602

[169] Mahlapuu M Håkansson J Ringstad L Björn C. Antimicrobial peptides: an emerging category of therapeutic agents. Front Cell Infect Microbiol (2016) 6:194. doi: 10.3389/fcimb.2016.00194 28083516 PMC5186781

[170] Zhang C Yang M. Antimicrobial peptides: from design to clinical application. Antibiotics (Basel) (2022) 11(3):349. doi: 10.3390/antibiotics11030349 35326812 PMC8944448

[171] Lin JS Bekale LA Molchanova N Nielsen JE Wright M Bacacao B. Anti-persister and anti-biofilm activity of self-assembled antimicrobial peptoid ellipsoidal micelles. ACS Infect Dis (2022) 8(9):1823–30. doi: 10.1021/acsinfecdis.2c00288 PMC9469094 36018039

[172] Amaral AC Silva ON Mundim NC de Carvalho MJ Migliolo L Leite JR. Predicting antimicrobial peptides from eukaryotic genomes: in silico strategies to develop antibiotics. Peptides (2012) 37(2):301–8. doi: 10.1016/j.peptides.2012.07.021 22884922

[173] Tomazou M Oulas A Anagnostopoulos AK Tsangaris GT Spyrou GM. In silico identification of antimicrobial peptides in the proteomes of goat and sheep milk and feta cheese. Proteomes (2019) 7(4):32. doi: 10.3390/proteomes7040032 31546575 PMC6958355

[174] Yakovlev IA Lysøe E Heldal I Steen H Hagen SB Clarke JL. Transcriptome profiling and in silico detection of the antimicrobial peptides of red king crab paralithodes camtschaticus. Sci Rep (2020) 10(1):12679. doi: 10.1038/s41598-020-69126-4 32728087 PMC7391757

[175] Porto WF Irazazabal LN Humblot V Haney EF Ribeiro SM Hancock REW. EcDBS1R6: a novel cationic antimicrobial peptide derived from a signal peptide sequence. Biochim Biophys Acta Gen Subj (2020) 1864(9):129633. doi: 10.1016/j.bbagen.2020.129633 32416198

[176] Raj S Venugopal U Pant G Kalyan M Arockiaraj J Krishnan MY. Anti-mycobacterial activity evaluation of designed peptides: cryptic and database filtering based approach. Arch Microbiol (2021) 203(8):4891–9. doi: 10.1007/s00203-021-02474-5 34244831

[177] Okella H Georrge JJ Ochwo S Ndekezi C Koffi KT Aber J. New putative antimicrobial candidates: in silico design of fish-derived antibacterial peptide-motifs. Front Bioeng Biotechnol (2020) 8:604041. doi: 10.3389/fbioe.2020.604041 33344436 PMC7744477

[178] Yin Q Wu S Wu L Wang Z Mu Y Zhang R. A novel in silico antimicrobial peptide DP7 combats MDR pseudomonas aeruginosa and related biofilm infections. J Antimicrob Chemother (2020) 75(11):3248–59. doi: 10.1093/jac/dkaa308 32737484

[179] Zhong C Zhu N Zhu Y Liu T Gou S Xie J. Antimicrobial peptides conjugated with fatty acids on the side chain of d-amino acid promises antimicrobial potency against multidrug-resistant bacteria. Eur J Pharm Sci (2020) 141:105123. doi: 10.1016/j.ejps.2019.105123 31676352

[180] Lu J Xu H Xia J Ma J Xu J Li Y. D- and unnatural amino acid substituted antimicrobial peptides with improved proteolytic resistance and their proteolytic degradation characteristics. Front Microbiol (2020) 11:563030. doi: 10.3389/fmicb.2020.563030 33281761 PMC7688903

[181] Pirtskhalava M Amstrong AA Grigolava M Chubinidze M Alimbarashvili E Vishnepolsky B. DBAASP v3: database of antimicrobial/cytotoxic activity and structure of peptides as a resource for development of new therapeutics. Nucleic Acids Res (2021) 49(D1):D288–97. doi: 10.1093/nar/gkaa991 PMC7778994 33151284

[182] D’Souza AR Necelis MR Kulesha A Caputo GA Makhlynets OV. Beneficial impacts of incorporating the non-natural amino acid azulenyl-alanine into the trp-rich antimicrobial peptide buCATHL4B. Biomolecules (2021) 11(3):421. doi: 10.3390/biom11030421 33809374 PMC8001250

[183] Yang M Zhang C Zhang MZ Zhang S. Novel synthetic analogues of avian beta-defensin-12: the role of charge, hydrophobicity, and disulfide bridges in biological functions. BMC Microbiol (2017) 17(1):43. doi: 10.1186/s12866-017-0959-9 28231771 PMC5324278

[184] Vicente FEM González-Garcia M Diaz Pico E Moreno-Castillo E Garay HE Rosi PE. Design of a helical-stabilized, cyclic, and nontoxic analogue of the peptide Cm-p5 with improved antifungal activity. ACS Omega (2019) 4(21):19081–95. doi: 10.1021/acsomega.9b02201 PMC6868880 31763531

[185] Khara JS Mojsoska B Mukherjee D Langford PR Robertson BD Jenssen H. Ultra-short antimicrobial peptoids show propensity for membrane activity against multi-drug resistant mycobacterium tuberculosis. Front Microbiol (2020) 11:417. doi: 10.3389/fmicb.2020.00417 32256474 PMC7089965

[186] Zuckermann RN. Peptoid origins. Biopolymers (2011) 96(5):545–55. doi: 10.1002/bip.21573 21184486

[187] Wang Z Liu X Da Teng Mao R Hao Y Yang N. Development of chimeric peptides to facilitate the neutralisation of lipopolysaccharides during bactericidal targeting of multidrug-resistant escherichia coli. Commun Biol (2020) 3(1):41. doi: 10.1038/s42003-020-0761-3 31974490 PMC6978316

[188] Moiola M Memeo MG Quadrelli P. Stapled peptides-a useful improvement for peptide-based drugs. Molecules (2019) 24(20):3654. doi: 10.3390/molecules24203654 31658723 PMC6832507

[189] Zhang SK Song JW Gong F Li SB Chang HY Xie HM. Design of an alpha-helical antimicrobial peptide with improved cell-selective and potent anti-biofilm activity. Sci Rep (2016) 6:27394. doi: 10.1038/srep27394 27271216 PMC4897634

[190] Wilde CG Griffith JE Marra MN Snable JL Scott RW. Purification and characterization of human neutrophil peptide 4, a novel member of the defensin family. J Biol Chem (1989) 264(19):11200–3. doi: 10.1016/S0021-9258(18)60449-1 2500436

[191] Yang Z He S Wu H Yin T Wang L Shan A. Nanostructured antimicrobial peptides: crucial steps of overcoming the bottleneck for clinics. Front Microbiol (2021) 12:710199. doi: 10.3389/fmicb.2021.710199 34475862 PMC8406695

[192] Deshayes C Arafath MN Apaire-Marchais V Roger E. Drug delivery systems for the oral administration of antimicrobial peptides: promising tools to treat infectious diseases. Front Med Technol (2021) 3:778645. doi: 10.3389/fmedt.2021.778645 35146486 PMC8821882

[193] Carratala JV Serna N Villaverde A Vázquez E Ferrer-Miralles N. Nanostructured antimicrobial peptides: the last push towards clinics. Biotechnol Adv (2020) 44:107603. doi: 10.1016/j.biotechadv.2020.107603 32738381

[194] Martin-Serrano A Gómez R Ortega P de la Mata FJ. Nanosystems as vehicles for the delivery of antimicrobial peptides (AMPs). Pharmaceutics (2019) 11(9):448. doi: 10.3390/pharmaceutics11090448 31480680 PMC6781550

[195] Patel A Cholkar K Mitra AK. Recent developments in protein and peptide parenteral delivery approaches. Ther Delivery (2014) 5(3):337–65. doi: 10.4155/tde.14.5 PMC4130463 24592957

[196] Sinha R Shukla P. Antimicrobial peptides: recent insights on biotechnological interventions and future perspectives. Protein Pept Lett (2019) 26(2):79–87. doi: 10.2174/0929866525666181026160852 30370841 PMC6416458

[197] Bakare OO Gokul A Fadaka AO Wu R Niekerk LA Barker AM. Plant antimicrobial peptides (PAMPs): features, applications, production, expression, and challenges. Molecules (2022) 27(12):3703. doi: 10.3390/molecules27123703 35744828 PMC9229691

[198] Ciura K Ptaszyńska N Kapica H Pastewska M Łęgowska A Rolka K. Can immobilized artificial membrane chromatography support the characterization of antimicrobial peptide origin derivatives? Antibiotics (Basel) (2021) 10(10):1237. doi: 10.3390/antibiotics10101237 34680817 PMC8532876

[199] Yang K Gitter B Rüger R Wieland GD Chen M Liu X. Antimicrobial peptide-modified liposomes for bacteria targeted delivery of temoporfin in photodynamic antimicrobial chemotherapy. Photochem Photobiol Sci (2011) 10(10):1593–601. doi: 10.1039/c1pp05100h 21773628

[200] Bruna T Maldonado-Bravo F Jara P Caro N. Silver nanoparticles and their antibacterial applications. Int J Mol Sci (2021) 22(13):7202. doi: 10.3390/ijms22137202 34281254 PMC8268496

[201] de Lacerda Coriolano D de Souza JB Bueno EV Medeiros SMFRDS Cavalcanti IDL Cavalcanti IMF. Antibacterial and antibiofilm potential of silver nanoparticles against antibiotic-sensitive and multidrug-resistant pseudomonas aeruginosa strains. Braz J Microbiol (2021) 52(1):267–78. doi: 10.1007/s42770-020-00406-x PMC7966632 33231865

[202] Jin Y Yang Y Duan W Qu X Wu J. Synergistic and on-demand release of Ag-AMPs loaded on porous silicon nanocarriers for antibacteria and wound healing. ACS Appl Mater Interfaces (2021) 13(14):16127–41. doi: 10.1021/acsami.1c02161 33787222

[203] Rai A Pinto S Velho TR Ferreira AF Moita C Trivedi U. One-step synthesis of high-density peptide-conjugated gold nanoparticles with antimicrobial efficacy in a systemic infection model. Biomaterials (2016) 85:99–110. doi: 10.1016/j.biomaterials.2016.01.051 26866877

[204] Yu CY Huang W Li ZP Lei XY He DX Sun L. Progress in self-assembling peptide-based nanomaterials for biomedical applications. Curr Top Med Chem (2016) 16(3):281–90. doi: 10.2174/1568026615666150701114527 26126914

[205] Yaghubi Kalurazi T Jafari A. Evaluation of magnesium oxide and zinc oxide nanoparticles against multi-drug-resistance mycobacterium tuberculosis. Indian J Tuberc (2021) 68(2):195–200. doi: 10.1016/j.ijtb.2020.07.032 33845951

